# Extracellular vesicles of *Lactiplantibacillus plantarum* PCM 2675 and *Lacticaseibacillus rhamnosus* PCM 489: an introductory characteristic

**DOI:** 10.20517/evcna.2024.49

**Published:** 2024-11-07

**Authors:** Katarzyna Kowalik, Kamila Kulig, Elzbieta Karnas, Olga Barczyk-Woznicka, Ewa Zuba-Surma, Elzbieta Pyza, Maria Rapala-Kozik, Justyna Karkowska-Kuleta

**Affiliations:** ^1^Department of Comparative Biochemistry and Bioanalytics, Faculty of Biochemistry, Biophysics and Biotechnology, Jagiellonian University, Kraków 30-387, Poland.; ^2^Doctoral School of Exact and Natural Sciences, Faculty of Biochemistry, Biophysics and Biotechnology, Jagiellonian University, Kraków 30-387, Poland.; ^3^Department of Cell Biology, Faculty of Biochemistry, Biophysics and Biotechnology, Jagiellonian University, Kraków 30-387, Poland.; ^4^Department of Cell Biology and Imaging, Institute of Zoology and Biomedical Research, Jagiellonian University, Kraków 30-387, Poland.

**Keywords:** Extracellular vesicles, EVs, probiotics, postbiotics, lactic acid bacteria

## Abstract

**Aim:** Extracellular vesicles (EVs) are involved in intercellular and interkingdom communication in the complex communities that constitute the niche-specific microbiome of the colonized host. Therefore, studying the structure and content of EVs produced by resident bacteria is crucial to understanding their functionality and impact on the host and other microorganisms.

**Methods:** Bacterial EVs were isolated by differential centrifugation, their size and concentration were measured by transmission electron microscopy and nanoparticle tracking analysis, and the cargo proteins were identified by liquid chromatography coupled to tandem mass spectrometry. The cytotoxicity of bacterial EVs was tested using the human epithelial cell line A549 and an *in vivo* model of *Galleria mellonella* larvae.

**Results:** The isolation and preliminary characteristics of EVs from two strains of lactic acid bacteria - *Lactiplantibacillus plantarum* PCM 2675 and *Lacticaseibacillus rhamnosus* PCM 489 - were presented, confirming the production of vesicular structures with sizes in the range of 50-170 nm for *L. plantarum* and 80-250 nm for *L. rhamnosus*. In addition, various proteins were identified within EVs cargo, with distinct locations of origin, including membrane, cytoplasmic and extracellular proteins, and with diverse functions, including enzymes with confirmed proteolytic activity. Furthermore, bacterial EVs did not show statistically significant cytotoxicity to the host under the tested conditions.

**Conclusions**: A better understanding of the composition and functionality of bacterial EVs may contribute to their future effective use in supporting human health.

## INTRODUCTION

Lactic acid bacteria *Lactiplantibacillus plantarum* and *Lacticaseibacillus rhamnosus*, being found naturally in fermented foods and residing in different niches of the host organism, exhibit a number of beneficial functions for human health and could therefore be considered probiotics^[1]^. As recent studies have shown, these species contribute to the maintenance of oral and gut health by interacting with other microorganisms in microbiome, inducing changes in the specific inhabited niche, competing with pathogens for surfaces and nutrients, releasing substances with antimicrobial properties, and modulating of the host immune system^[2,3]^.

Considering the alteration of the balance in different niches of the host organism, several studies have been conducted recently on the impact of *L. plantarum* and *L. rhamnosus* on other residing microorganisms. In the case of microorganisms inhabiting the first part of the digestive system, that is, the oral cavity, the expression of virulence-related genes and the formation of biofilms by bacteria *Streptococcus mutans* and fungi *Candida albicans* - key pathogens responsible for different disorders in the mouth, including dental caries and oral thrush - are affected by *L. plantarum* strain 14917^[4,5]^. In addition, plantaricin, an antimicrobial peptide produced by this strain, inhibited the growth of these pathogenic microorganisms^[6]^. In other studies, *L. plantarum* VHProbi^®^ V38 demonstrated antimicrobial activity against not only *S. mutans*, but also periodontopathogens *Porphyromonas gingivalis*, *Aggregatibacter actinomycetemcomitans*, and *Fusobacterium nucleatum*, strongly reducing their adhesion to human primary gingival epithelial cells^[7]^. Likewise, for *L. rhamnosus* VHProbi^®^ M14, the inhibition of *C. albicans*, as well as a broad spectrum of oral bacteria, has been shown, including *S. mutans*, *F. nucleatum*, *A. actinomycetemcomitans*, and *P. gingivalis*^[8]^. Both probiotic strains also exhibited significant antibiofilm activity targeting oral pathogens^[9]^.

Additionally, studies on the mechanisms of the regulation of intestinal microbiota have shown that *L. plantarum* stimulates the growth of bacteria that are beneficial to the host and inhibit the harmful species^[10]^. *L. plantarum* ZFM4 provided a protective effect during infection caused by *Helicobacter pylori*^[11]^, while exopolysaccharide produced by *L. plantarum* Y12 diminished pathogenicity and biofilm formation by enteric pathogen *Shigella flexneri*^[12]^. *L. rhamnosus* also demonstrated the properties of inhibiting the colonization and growth of pathogenic microorganisms, including staphylococci and streptococci, in different host niches such as the intestinal tract or skin^[13]^. Therefore, *L. plantarum* and *L. rhamnosus* are considered particularly important species involved in ensuring the microbiological balance in the host organisms and providing additional protection against microbial infections^[14]^. A range of other beneficial effects on host cells exhibited by these species, including their antioxidative capacity, interactions with host immune cells resulting in the production of proinflammatory and anti-inflammatory cytokines, induction of IgA production, and upregulation of Toll-like receptors (TLRs) made *L. plantarum* and *L. rhamnosus* important components of the regulatory machinery responsible for the maintenance of human health^[15,16]^.

Currently, postbiotics specified as inanimate probiotic microorganisms, their components or products including bacteriocins, lactic acid, hydrogen peroxide, and others attract significant attention due to their health-promoting potential for use, while reducing the risk associated with the overgrowth of viable microbial cells, development of infections in immunocompromised individuals, or horizontal gene transfer from probiotics to other bacteria contributing to the spread of antibiotic resistance^[17-20]^. As demonstrated recently in numerous studies, postbiotics might be involved in inhibiting pathogenic bacteria and in immunomodulatory activities, resulting in beneficial effects for host and regulation of the inflammatory processes^[21]^. Postbiotics from *L. rhamnosus* GG were capable of inhibiting *S. mutans* biofilm, metabolic activity, and expression of gene *gtfB*^[22]^, while postbiotics from *L. plantarum* PD18 prevented biofilm formation by *S. mutans* and *P. gingivalis*^[23]^. *L. plantarum* postbiotics also suppressed the pathogenicity of *Salmonella enterica* Typhimurium by reducing the expression of virulence-related genes, pili- and flagellum-encoding genes, and biofilm formation-related genes^[24]^. *L. plantarum* and *L. rhamnosus* postbiotics inhibited the viability and metabolic activity of fungi *Candida parapsilosis*, also affecting interactions of fungal cells with vaginal epithelium^[25]^. Therefore, not only live bacterial cells, but also their products and derivatives may be beneficial in the maintenance of healthy balance in inhabited niches, including the oral cavity, further sections of the gastrointestinal tract, or the vagina^[9,20,25]^. This group of particles includes bacterial extracellular vesicles (EVs) - nanometer-sized structures enclosed with a lipid bilayer and containing a wide variety of biologically active molecules. To date, a wide range of functions of probiotic EVs have been demonstrated to support host health through various mechanisms. The specific roles of EVs produced by probiotics in interspecies and interkingdom communication, and their impact on various physiological and pathological processes have been summarized and detailed in recent comprehensive reviews^[17,26,27]^. Numerous pieces of evidence have demonstrated that these structures can modify the environment colonized by probiotics through the action of transferred different molecules (e.g., antibacterial peptides, enzymes, organic acids) so as to inhibit the growth of pathogenic microorganisms and reduce the possibility of their overgrowth and colonization of the particular host niche^[17]^. Moreover, probiotic EVs may support the functioning of the intestinal epithelial barrier by strengthening its stability and influencing intercellular junctions (EVs are internalized by cells via clathrin-dependent endocytosis), and they modulate the activity of the innate and adaptive immunity by regulating nuclear factor kappa B (NFκB) levels and affecting the secretion of proinflammatory and anti-inflammatory cytokines^[17,27]^. The proposed mechanism of the influence of probiotic EVs on immune system cells may involve the internalization of vesicles by dendritic cells, mediated via interactions with TLRs TLR2 and TLR4, followed by the activation of intracellular signaling and activation of mitogen-activated protein kinases (MAPKs) pathway, and, in consequence, differentiation of naïve T-helper cells (Th0) into regulatory T cells (Treg) that produce interleukins IL-4, IL-10, and IL-22 with an anti-inflammatory mode of action; however, in other reports, the increase in proinflammatory cytokines tumor necrosis factor-alpha (TNF-α), IL-6, and IL-8 has also been described in the presence of probiotic EVs under varying host cell states and microenvironmental conditions^[26,27]^. Therefore, the currently available data, which still require extensive expansion, indicate that the mechanisms of action of probiotic EVs are complex and context-dependent. These mechanisms are influenced by the host immune system status, microbiota balance, and the presence of pathogenic microorganisms or their EVs, and are also determined by the specific species or probiotic strain involved. Consequently, these factors together may either suppress or enhance the organism’s defense response, aiming to fight adverse conditions and improve overall health.

EVs are pivotal for intercellular communication and their potential use as postbiotics is currently attracting research attention due to the greater safety of postbiotics in individuals with impaired immunity, compared to the use of live bacterial cells^[26,28]^. This requires an understanding of the conditions of effective production of EVs and an assessment of their non-toxicity after contact with host. Thus, the primary aims of this short communication are (i) to isolate EVs produced by two tested bacterial strains from cultures on solid media; (ii) to characterize the morphology and sizes of EVs and conduct an initial evaluation of their proteinaceous cargo; and (iii) to perform preliminary verification of potential adverse effects on the host in selected *in vitro* and *in vivo* models.

## METHODS

### Bacterial strains and growth conditions

*Lactiplantibacillus plantarum* PCM 2675 and *Lacticaseibacillus rhamnosus* PCM 489 (ATCC 9595) were purchased from Polish Collection of Microorganisms (Wroclaw, Poland) and cultivated in 20 mL of liquid De Man Rogosa and Sharp medium (MRS; Thermo Fisher Scientific, Waltham, MA, USA) in the microaerophilic atmosphere for 24 h at 37 °C, without shaking. The number of cells in liquid culture was estimated by measuring the optical density at 600 nm (OD_600_).

### EVs isolation

For isolation of EVs, 4.5 × 10^9^ bacterial cells were transferred to MRS agar plate [MRS broth with 1.5% (w/v) agar] and cultured for 24 h at 37 °C in an atmosphere of 5% CO_2_. Then, bacterial cells were carefully collected from the surface of the solid medium and transferred with sterile loop to Eppendorf tube containing 1 mL Dulbecco’s phosphate-buffered saline (DPBS), pH 7.50  ±  0.30 (Biowest, Nuaillé, France), and incubated for 10 min at room temperature. The number of viable bacterial cells producing EVs was assessed by transferring 10 µL from a series of ten-fold diluted bacterial cell suspensions, to a sterile MRS agar plate, followed by the incubation for 24 h at 37 °C and colony forming unit (CFU) counting. The next step in EVs separation from bacterial cells was the centrifugation for 10 min, 5,000 × g at 4 °C to sediment bacterial cells; after that, supernatants with EVs were transferred to the new Eppendorf tubes and further centrifuged twice for 15 min, 5,000 × g at 4 °C to remove any cells remnants. After discarding the sediment, collected supernatants were filtered using Ultrafree-CL Centrifugal Filter Unit (Merck, Darmstadt, Germany) with a pore size of 0.22 μm and transferred to sterilized polycarbonate thick wall centrifuge tubes (13 mM ×  64 mM) with 13-mM-diameter Delrin tube adapters, ultracentrifuged at 4 °C for 1 h with a relative centrifugal field of 144,000  × g (*k* factor 112) using a fixed-angle type 60 Ti Rotor in an Optima LE-80 K Ultracentrifuge (Beckman Coulter, Brea, CA, USA). Obtained EVs pellets were suspended in 400 μL of sterile DPBS and stored at -80 °C. After each isolation, 10 µL of the obtained sample containing EVs was transferred to a sterile MRS agar medium to confirm the absence of contamination with bacterial cells after incubation for 24 h at 37 °C. The nomenclature of the obtained vesicles is as follows - LpEVs for EVs produced by *L. plantarum* and LrEVs for EVs produced by *L. rhamnosus*.

### Protein and lipid concentration measurements

Protein concentrations in the EVs-containing samples were assessed using *o*-phthalaldehyde (OPA; Sigma-Aldrich, St. Louis, MO, USA)^[29]^. Briefly, 10 µL of sample and 300 µL of OPA reagent were applied to the wells of a black 96-well microplate (Greiner Bio-One, Kremsmünster, Austria) in six biological replicates, and after 5 min of incubation in the dark, the fluorescence intensity measurements were carried out at an excitation wavelength of 340 nm and emission of 450 nm using a Synergy H1 microplate reader (BioTek Instruments, Winooski, VT, USA). The phospholipid concentration in the EVs-containing samples was measured in three biological replicates using 20 µL of obtained sample of EVs of each bacterial species using the Phospholipid Assay Kit (Sigma-Aldrich) in accordance with the manufacturer’s instructions.

### Transmission electron microscopy imaging and nanoparticle tracking analysis measurements

Visualization of EVs was performed with transmission electron microscopy (TEM) with the use of JEOL JEM-2100 HT microscope (JEOL, Tokyo, Japan). Formvar-coated, 300 mesh copper grids were prepared for each vesicle sample by applying 7 µL of EVs suspension in DPBS buffer and allowing it to be adsorbed, and then negative staining using 2% uranyl acetate was performed (Chemapol, Prague, Czech Republic). The TEM images were acquired using a 4 k × 4 k camera (TVIPS) equipped with the EMMENU software version 4.0.9.87. Information on the size and concentration of the bacterial EVs was obtained using nanoparticle tracking analysis (NTA). Prepared samples were measured in a flow mode using NanoSight NS300 system, Blue488 laser, and NTA software Version 3.4 (Malvern Instruments, Malvern, UK). Each sample diluted in 0.22 µM-filtered DPBS, pH 7.50 ± 0.30 (Lonza, Basel, Switzerland) was recorded at 24 °C three times for 60 s with camera type scientific complementary metal-oxide-semiconductor (sCMOS) adjusted to level 13 and the threshold parameter set on 2.

### Proteomic identification of vesicular proteins

Identification of proteins contained in bacterial EVs was performed with liquid chromatography-tandem mass spectrometry (LC-MS/MS) and UltiMate 3000 RSLCnano System coupled with Q-Exactive mass spectrometer (Thermo Fisher Scientific, Waltham, MA, USA) with DPV-550 Digital PicoView nanospray source (New Objective, Woburn, MA, USA) as described in details previously^[29-31]^ with some minor modifications. Briefly, a sample containing EVs with a total amount of vesicular proteins equal to 20 µg was prepared in 100 μL of 100 mM Tris-HCl buffer, pH 7.60 ± 0.10, with 1% (w/v) sodium dodecyl sulfate. Then, the prepared mixture was sonicated in four cycles of 30 s each, using UP50H Compact Lab Homogenizer with 50 W, 30 kHz, amplitude 80%, cycle 0.5 (Hielscher Ultrasonics, Teltow, Germany), incubated for 5 min at 95 °C, and centrifuged for 12 min at 12,000 × g. After that, proteins were precipitated overnight with trichloroacetic acid at -20 °C, centrifuged at 10,000 × g for 15 min at 10 °C, and washed twice with ice-cold acetone. The obtained precipitate was dissolved in 100 μL of 10 mM N-2-hydroxyethylpiperazine-N’-2-ethanesulfonic acid (HEPES) buffer, pH 8.50 ± 0.20 and digested with 30 µL of 0.027 µg/µL Trypsin/Lys-C Mix (Promega, Mannheim, Germany). The obtained and separated peptides were identified after processing of obtained RAW files by the Proteome Discoverer platform (v.1.4.1.14, Thermo Fisher Scientific) and searched using a locally installed MASCOT search engine (v.2.5.1, Matrix Science, London, UK) and corresponding protein databases with taxonomy restrictions SwissProtTrEMBL_Lactobacillus_plantarum (25349 sequences) or SwissProtTrEMBL_Lactobacillus_ rhamnosus (15335 sequences) and NCBInr database (26490256 sequences) with taxonomy restriction Other Firmicutes (3598679 sequences). The search parameters were applied as follows: fixed modification - cysteine carbamidomethylation; variable modifications - methionine oxidation; precursor mass tolerance - 10 ppm; fragment mass tolerance - 20 mmu, target False Discovery Rate (Strict) - 0.01, maximum missed cleavage sites - 1. Two biological replicates for each species were prepared. Only proteins with scores above 20 were listed in Supplementary Materials. The mass spectrometry proteomics data have been deposited to the ProteomeXchange Consortium via the PRoteomics IDEntifications database (PRIDE) partner repository^[32]^ with the dataset identifier PXD048874 (*L. plantarum*) and PXD048876 (*L. rhamnosus*).

### Measurement of hydrolytic activity of EVs

The proteolytic activity of EVs was measured according to the procedure described previously^[31]^ using EnzChek™ Protease Assay Kit (Thermo Fisher Scientific) following the manufacturer’s instructions. Bacterial EVs (5 × 10^9^) were incubated in 100 µL of DPBS, pH 7.50 ± 0.30 in the wells of a black, flat-bottom 96-well microplate (Greiner Bio-One) with 1 μg of BODIPY FL casein at 37 °C in the dark for 24 h. The control was BODIPY FL casein in DPBS only, without EVs. Two biological replicates with three technical replicates each were performed. The fluorescence intensity was measured with excitation and emission wavelengths of 485 and 528 nm, respectively, with a Synergy H1 microplate reader.

### A549 cell culture

The A549 human lung adenocarcinoma cell line (CLL-185™) was cultured in F-12K medium (Kaighn’s modification of Ham’s F-12 medium) (Corning, New York, NY, USA) supplemented with 10% fetal bovine serum (FBS) (Gibco, Thermo Fisher Scientific), 100 U/mL penicillin, and 100 mg/mL streptomycin (Biowest) at 37 °C in the atmosphere of 5% CO_2_ and 95% humidity.

### XTT and Lactate Dehydrogenase release assays

Human cells from the cell line A549 (2 × 10^4^ cells) were incubated in the wells of 96-well flat base, polystyrene microplate with standard surface (Sarstedt, Nümbrecht, Germany) with 5 × 10^8^ EVs in 100 µL of F-12K medium for 24 h, then supernatants were removed, and cells were washed twice with 200 μL DPBS. To determine cell metabolic activity, the XTT (sodium 3′-[1-(phenylaminocarbonyl)-3,4-tetrazolium]-bis (4-methoxy6-nitro) benzene sulfonic acid hydrate) reduction test was performed, and 100 μL of RPMI 1640 medium without phenol red (Biowest) and 50 μL of XTT reagent [XTT at a final concentration of 1 mg/mL (Thermo Fisher Scientific) and PMS (N-methyl dibenzopyrazine methyl sulfate) at a final concentration of 3 µg/mL (Sigma)] were added for 1 h at 37 °C in the atmosphere of 5% CO_2_. To assess lactate dehydrogenase (LDH) release by A549 cells, analysis with Cytotoxicity Detection KitPLUS (LDH) (Roche, Basel, Switzerland) was performed according to the manufacturer’s instructions. Briefly, 50 µL of mix reagent (mix of 250 µL of reconstituted catalyst with 11.25 mL dye solution) was added. For positive control, 5 µL of lysis solution was added to the cells. After incubation for 15 min at 37 °C in the atmosphere of 5% CO_2_ and in the dark, the reaction was stopped by adding 25 µL of stop solution. Three biological replicates were performed. After transferring the supernatants to a new microplate (Sarstedt), the absorbance measurement was performed at 450 nm (XTT) or 490 nm (LDH) using a Synergy H1 microplate reader.

### *Galleria mellonella* survival

The *in vivo* safety of bacterial EVs was evaluated using *Galleria mellonella* moth larvae. For each group, ten randomly selected representative individuals in their last instar were chosen. Using 10 µL Hamilton syringe (Merck), 10 µL of samples contained 10^8^ or 10^9^ bacterial EVs in sterile DPBS buffer, pH 7.50 ± 0.30 (Biowest) were administered in the left last proleg of each larva. Survival at 37 °C was monitored for the next 7 days. Four or three biological replicates were performed for LpEVs or LrEVs, respectively.

### Statistical analysis

To analyze the statistical significance, an unpaired *t*-test or one-way ANOVA with Dunnett’s multiple comparisons test (for data shown as bar graphs) and Log-rank (Mantel-Cox) test (for survival rate analysis) were performed with GraphPad Prism software version 10.0.3 (GraphPad Software, La Jolla, CA, USA). Results on bar graphs are presented as mean ± standard deviation. Statistical significance levels versus control were indicated by ^****^ for *P* < 0.0001, and ns when not significant.

## RESULTS

**Figure 1:**
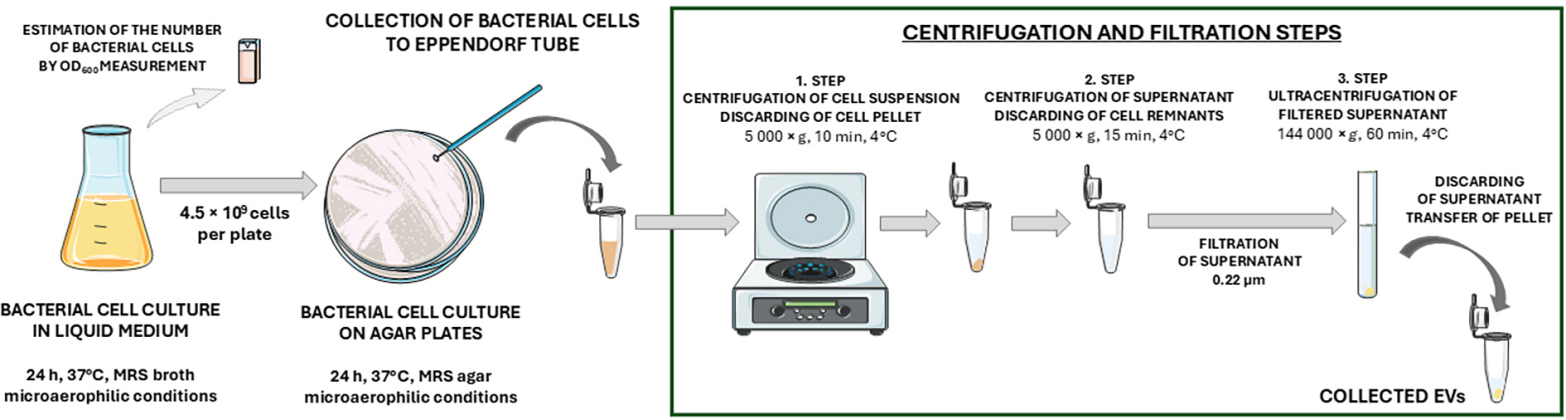
Step-by-step procedure for isolation of bacterial EVs from cells grown on MRS solid media. The figure was partly generated using Servier Medical Art, provided by Servier, licensed under a Creative Commons Attribution 4.0 unported license. EVs: Extracellular vesicles; MRS: De Man Rogosa and Sharp.

Bacterial EVs were isolated from cultures on solid media using a previously described method^[29]^. Briefly, bacterial strains *L. plantarum* PCM 2675 and *L. rhamnosus* PCM 489 (ATCC 9595) were first cultivated at 37 °C under microaerophilic conditions in MRS liquid medium, and after 24 h, each culture was diluted a hundred times, resulting in OD_600_ of 0.95 for *L. plantarum* and 0.39 for *L. rhamnosus*. Subsequently, 54 and 130 µL of each diluted culture were transferred to MRS agar plates for further growth under the same conditions. To minimize the accumulation of the sedimented and concentrated liquid medium components in the obtained EVs-containing samples, only bacterial cells were collected from the solid medium. In addition, after each isolation, it was confirmed that the obtained EV samples did not contain any viable bacterial cells. The procedure for isolating bacterial EVs from cultures on solid media is schematically presented in Figure 1.

**Figure 2:**
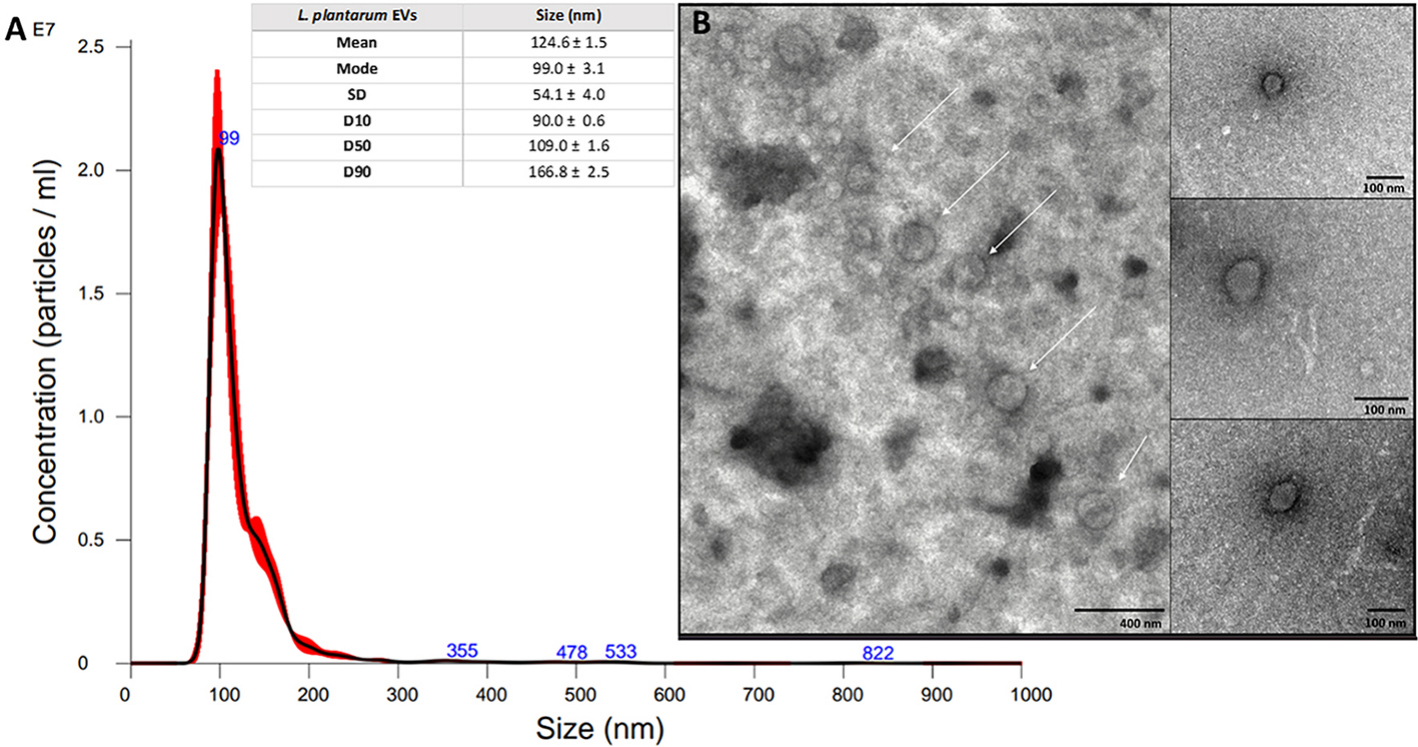
Characteristics of EVs produced by *L. plantarum* PCM 2675. (A) NTA-based particle size distribution analysis; a representative histogram of the average size distribution from three measurements of a single sample (black line) is presented. The presented blue numbers indicate the maxima of peaks, and the red areas show the SD between measurements. The size parameters of EVs are included. Factors D10, D50, and D90 denote that 10%, 50%, and 90% of the EV population had a diameter of less than or equal to the presented value; (B) TEM images of LpEVs. White arrows point to the individual particles. Scale bars included in individual panels. EVs: Extracellular vesicles; NTA: nanoparticle tracking analysis; TEM: transmission electron microscopy; SD: standard deviation.

**Figure 3:**
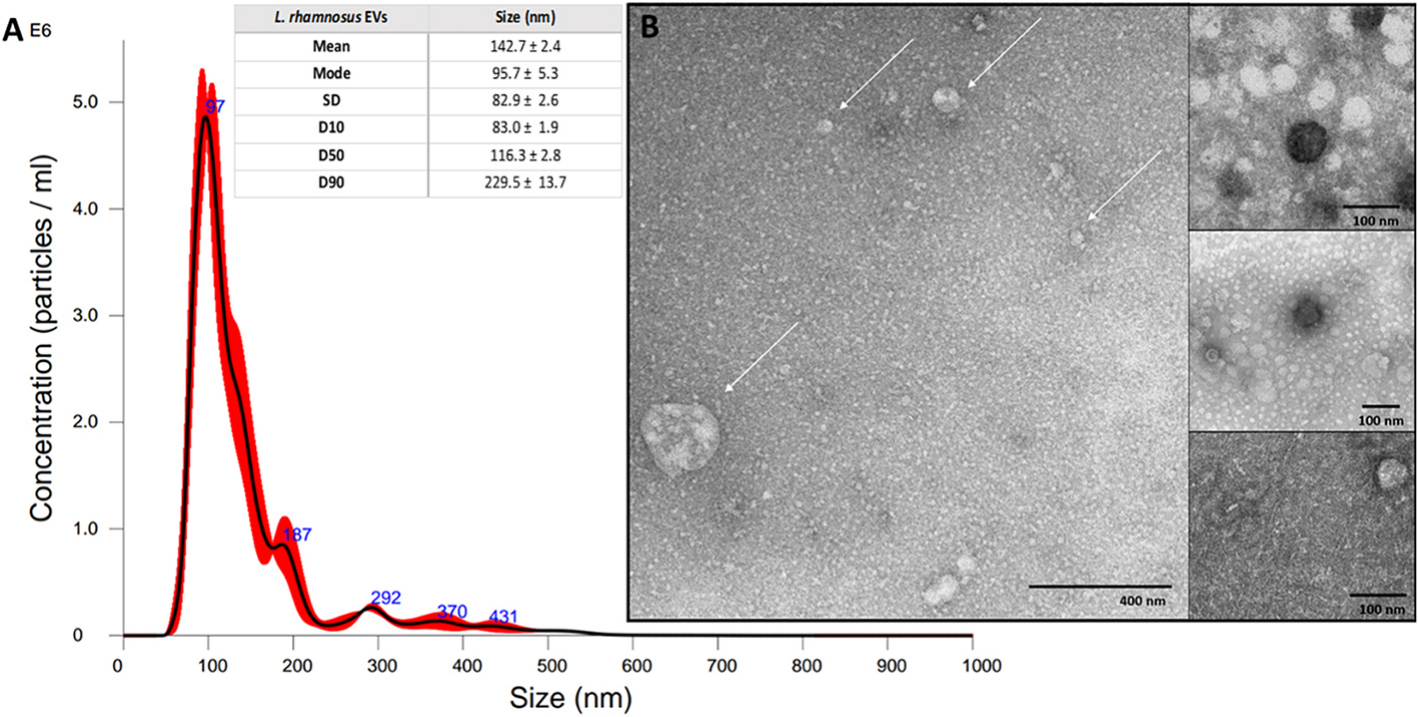
Characteristics of EVs from *L. rhamnosus* PCM 489. (A) NTA-based particle size distribution analysis; a representative histogram of the average size distribution from three measurements of a single sample (black line) is presented. The presented blue numbers indicate the maxima of peaks, and the red areas show the SD between measurements. The size parameters of EVs are included. Factors D10, D50, and D90 denote that 10%, 50%, and 90% of the EV population had a diameter of less than or equal to the presented value; (B) TEM images of LrEVs. White arrows point to the individual particles. Scale bars included in individual panels. EVs: Extracellular vesicles; NTA: nanoparticle tracking analysis; TEM: transmission electron microscopy; SD: standard deviation.

NTA-based size distribution measurement of isolated particles indicated a mode value of 99.9 ± 3.1 nm for EVs produced by *L. plantarum* [Figure 2A], and 95.7 ± 5.3 nm for *L. rhamnosus* [Figure 3A]. The number of vesicles produced by the detected number of living bacterial cells collected from one MRS agar plate has been assessed based on particle concentration measurement with NTA and on CFU counting. Accomplished estimations indicated that 2.86 × 10^12^ viable *L. plantarum* cells enabled the isolation of about 2.58 × 10^11^ EVs, and for 1.08 × 10^12^ viable *L. rhamnosus* cells, about 3.66 × 10^10^ particles were isolated. Using TEM, the presence and morphology of spherical structures in obtained samples were confirmed for *L. plantarum* [Figure 2B] and *L. rhamnosus* [Figure 3B]. Notably, some heterogeneity in EVs populations was observed, particularly for *L. rhamnosus*. Furthermore, the presence of structures composed of lipids and proteins was also confirmed by the measurement of their average concentration in the obtained EVs-containing samples. The average phospholipid concentration was 17 ± 1.6 µM for LpEVs and 8.8 ± 0.5 µM for LrEVs, while the average protein concentration was 0.93 ± 0.15 mg/mL and 1.06 ± 0.16 mg/mL for LpEVs and LrEVs, respectively.

**Figure 4:**
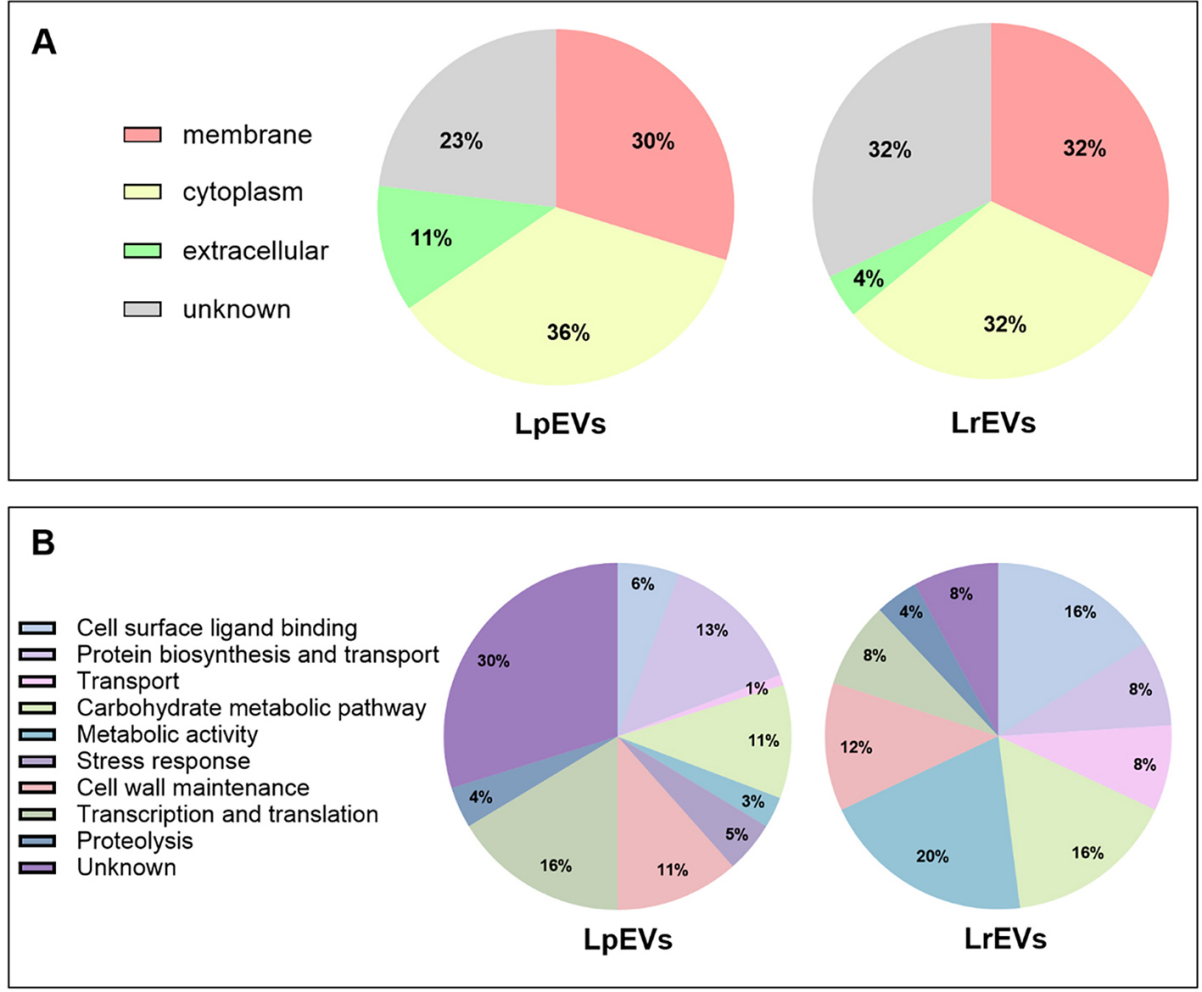
Characteristics of proteins identified in LpEVs and LrEVs. (A) Proteins grouped by their location; (B) Proteins grouped by their function.

Subsequently, proteomic identification of proteins found in bacterial vesicles was performed in duplicate with the shotgun proteomic approach and LC-MS/MS. Identifying these proteins and indicating their functions might not only elucidate the mechanisms by which probiotic EVs interact with other cells, but also imply potential biogenesis pathways, and enable the projection of further application of EVs across various fields. The analyses showed some discrepancy in the number of identified proteins in both samples, despite the same protein concentration. For LpEVs and LrEVs, the total amount of protein subjected to analysis was the same and the obtained mass spectra had similar intensities, so the differences in the number of identified proteins most likely result from the available content of the databases for both tested species. For *L. plantarum*, all identified proteins are listed in Supplementary Table 1, while for *L. rhamnosus* in Supplementary Table 2, and for further comparisons, proteins identified in both replicates were selected. The analysis of the location and functions of identified vesicular proteins [Figure 4] was performed by assigning specific annotations to individual molecules based on data included in the UniProt protein database (https://www.uniprot.org/)^[33]^ and InterPro protein families database (https://www.ebi.ac.uk/inter pro/)^[34]^.

In the case of EVs of both species tested, primarily membrane (30% for LpEVs and 32% for LrEVs) and cytoplasmic (36% for LpEVs and 32% for LrEVs) proteins were detected among proteins with known locations; additionally, molecules with an assigned extracellular location were also identified (11% for LpEVs and 4% for LrEVs) [Figure 4A]. That would confirm the origin of EVs as extracellular structures originating from the cell, surrounded by a membrane, and transporting some of the intracellular proteins^[35]^. Classification of proteins based on their function assignment showed that vesicular proteins identified for *L. plantarum* are involved mostly in protein biosynthesis and transport (13%), transcription and translation (16%), cell wall maintenance (11%), carbohydrate metabolism (11%), while for *L. rhamnosus* in the cell wall biosynthesis and remodeling (12%), carbohydrate metabolism (16%) and other metabolic pathways (20%) [Figure 4B]. Proteins involved in the transport of distinct types of molecules (1% for LpEVs or 8% for LrEVs) and in the interactions with ligands at the cell surface (6% for LpEVs or 16% for LrEVs) were also identified for both species. Taking into consideration proteins responsible for transport, for LpEVs, adenosine triphosphate-binding cassette (ABC)-type oligopeptide transport system, amino acid ABC transporters, bacteriocin transport accessory protein, manganese transport protein MntH have been identified, and for LrEVs, ABC transporter substrate-binding protein, oligopeptide ABC transporter substrate-binding protein, calcium ABC transporter adenosine triphosphate (ATPase), phosphotransferase system (PTS sugar transporter), and ABC transporter permease have been found in EVs. Among the proteins involved in metabolism, lactate dehydrogenase, glyceraldehyde-3-phosphate dehydrogenase, enolase, and glucose-6-phosphate isomerase have been identified in EVs of both tested species, and fructose-1,6-bisphosphate aldolase, phosphoglycerate kinase and pyruvate kinase in LpEVs, and tagatose 1,6-diphosphate aldolase in LrEVs.

**Figure 5:**
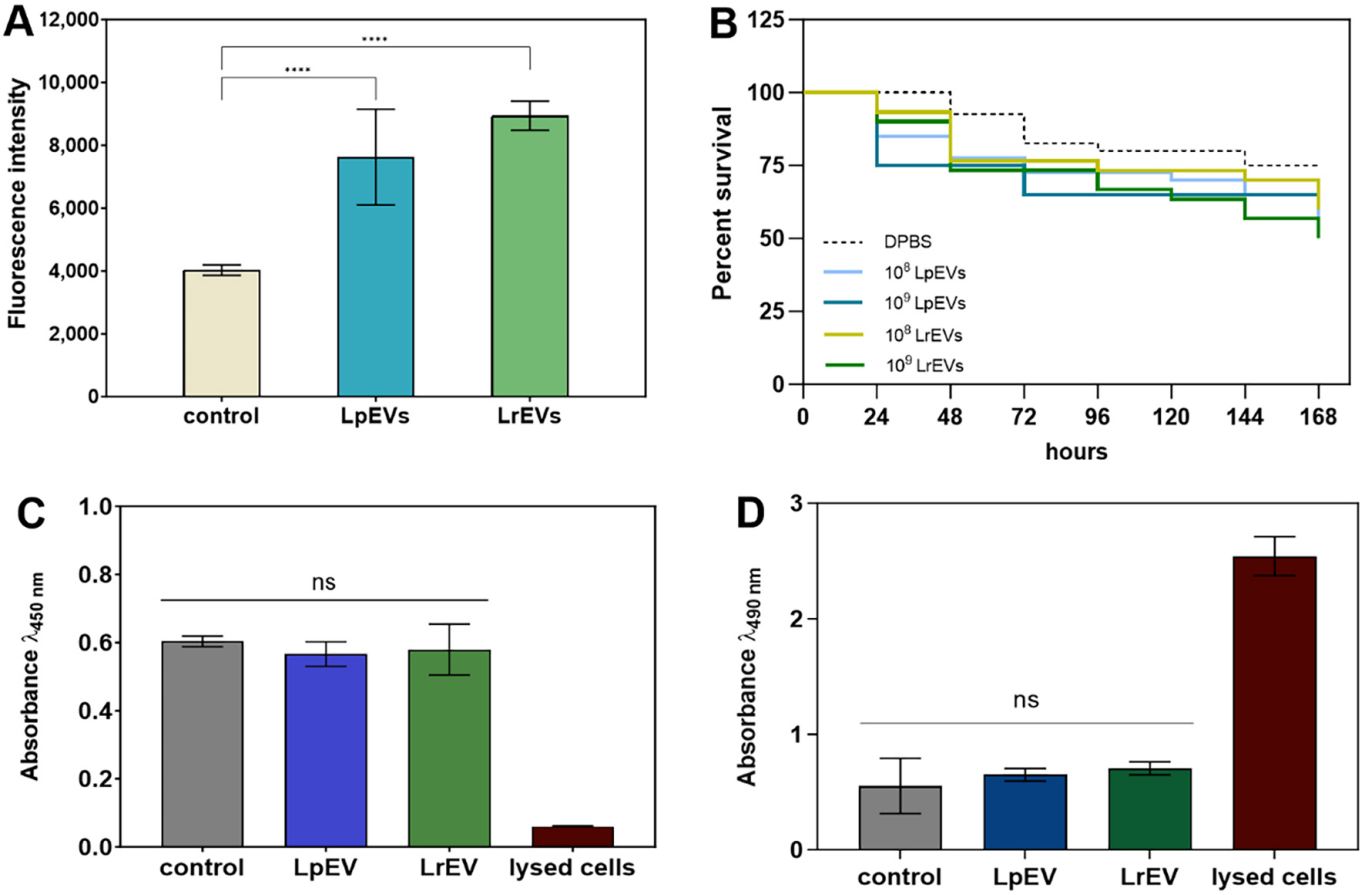
The effect of bacterial EVs on the host. (A) EVs-related proteinase activity measured with BODIPY FL casein as a substrate. The control sample was DPBS with substrate, without EVs. Statistical significance levels versus control are marked as ^****^ for *P* < 0.0001, and ns when not significant; (B) The survival curve of *Galleria mellonella* larvae after the injection of EVs produced by *L. plantarum* (LpEV) and *L. rhamnosus* (LrEV). Injection with DPBS served as a control; (C) The metabolic activity and (D) LDH release by A549 epithelial cells following treatment with 5 × 10^8^ EVs produced by *L. plantarum* (LpEV) and *L. rhamnosus* (LrEV). Untreated epithelial cells and lysed cells served as negative and positive controls, respectively. Statistical significance levels versus control: ns when not significant. Evs: Extracellular vesicles; DPBS: Dulbecco’s phosphate-buffered saline.

The proteomic identification of vesicular proteins indicated the presence of various enzymes within bacterial EVs, prompting further investigation into the proteolytic activity of these EVs. Among the identified enzymes in LpEVs were extracellular zinc metalloproteinase Zmp2, peptidase Do, dipeptidase PepV, serine-type D-Ala-D-Ala carboxypeptidase, and in LrEVs, carboxypeptidase, aminopeptidase, endopeptidase La, S8 family serine peptidase, serine-type D-Ala-D-Ala carboxypeptidase, and ATP-dependent zinc metalloprotease FtsH [Supplementary Tables 1 and 2]. For both tested species, the noticeable ability to hydrolyze peptide bonds has been demonstrated with the use of casein as a proteinaceous substrate [Figure 5A].

Since, in addition to examining the composition of EVs, it is also critical to verify their impact on the host and test whether they are non-toxic to host cells, an *in vitro* model using human epithelial cells and an *in vivo* system with the model organism *Galleria mellonella* were used for this purpose. The influence of intra-hemocoel injection of LpEVs or LrEVs on the survival of *G. mellonella* larvae was tested for 7 days after application of EVs at two concentrations - 10^8^ and 10^9^ prepared in 10 µL of DPBS buffer per larva [Figure 5B]. In the case of both types of EVs and both concentrations used, there was no statistically significant difference in the survival rate of larvae when comparing all groups analyzed (*P* = 0.3109 with Log-rank Mantel-cox test). Some noticeable, but statistically not significant decrease in larvae survival was observed over the course of the experiment for groups injected with different EVs concentrations (10^8^ *vs*. 10^9^ per larva) with lower survival rates at higher EVs concentrations (for LpEVs *P* = 0.9133 and for LrEVs *P* = 0.4103). When compared to DPBS-injected larvae, the group injected with 10^9^ EVs also showed slightly reduced survival, but the differences again were statistically not significant (*P* = 0.1120 for LpEVs and *P* = 0.1364 for LrEVs).

Moreover, the effect of EVs released by both bacterial species on human epithelial cell line A549 was tested with the XTT assay and with the measurement of LDH release [Figure 5]. The metabolic activity of A549 cells after incubation with LpEVs and LrEVs demonstrated a slight decrease, but without statistical significance [Figure 5C]. Furthermore, the measurement of LDH release, correlating with the level of the cell damage, indicated the comparable level of released LDH after treatment of cells with bacterial EVs compared to the control cells, as the minor changes were statistically not significant [Figure 5D].

## DISCUSSION

The knowledge of EVs production by Gram-positive bacteria is still incomplete and the mechanisms of their biogenesis and release require intensive investigation, as they differ from those reported for Gram-negative bacteria, also due to the presence of a thick layer of cell wall peptidoglycan on the bacterial cell surface^[36]^. Thus far, it has been indicated that EVs released by Gram-positive bacteria may have various biogenesis pathways, often accompanied by bacterial cell wall degradation, and based on membrane blebbing and explosive cell lysis^[37]^. This may also result in significant heterogeneity of these structures, considering not only their sizes, but also composition and properties. In general, the isolation of bacterial EVs also presents challenges that necessitate optimization for both efficiency and contamination-free preparations. Purification of EVs from liquid cultures may pose a risk of co-isolation of the medium components concentrated together with vesicles^[38]^. Therefore, in the case of other microorganisms, an improved method of EV isolation has recently been introduced, which involves EVs isolation from fungal cells cultured on solid media. This approach minimized the risk of co-concentration of the medium components, since only microbial cells were collected from the surface of plates containing solid media, and then suspended in a phosphate-buffered saline. In addition, the noteworthy advantages of this method, compared to isolation from a liquid medium, include significantly reduced procedural time and decreased labor intensity. Thus, the procedure of isolating EVs from solid media cultures has been considered fast, reproducible, and reliable^[39]^. Therefore, in this work, we proposed the use of this method for the isolation of EVs produced by two species of Gram-positive bacteria that are classified as probiotic microorganisms, and we presented their preliminary characteristics to provide an introduction to further advanced research on their functionality as postbiotics. The pleiotropic effects of postbiotics from lactic acid bacteria currently attract significant interest. Their wide application range includes nutritional support, modulation of the host immune response, anticancer activity, and antimicrobial action against different pathogenic microorganisms^[17,26,27,40-44]^. EVs produced by *Lactobacillaceae*, considered as bioactive compounds, fit into the criteria established for postbiotics, and their beneficial effects, often comparable to those of viable bacteria, have been repeatedly demonstrated^[45,46]^. Therefore, a more detailed knowledge about EVs may contribute to a better understanding of their application possibilities, and some interesting studies with physicochemical characteristics and proteomic analysis of EVs produced by different lactobacilli have been recently published^[47-52]^.

In this work, EVs from two strains from this family - *Lactiplantibacillus plantarum* PCM 2675 and *Lacticaseibacillus rhamnosus* PCM 489 - were isolated and characterized. Their sizes correspond to those specified for other strains, although the reported ranges are quite variable. EVs produced by *L*. *plantarum* strain WCFS1 were in the range of 31-200 nm^[47]^, for strain KCTC 11401BP 20-100 nm^[53]^, and for strain BGAN8 20-140 nm^[48]^ with the average size of about 50 nm as a monomer and about 800 nm as a multimeric structure^[49]^. For EVs derived from *L. plantarum* Q7, their sizes ranged from 70 nm to 500 nm with a mean size of 185.5 ± 65.4 nm^[54]^, and the mean size of EVs isolated from *L. plantarum* APsulloc 331261 was 104 nm^[55]^. Studies describing EVs produced by *L. rhamnosus* GG strain reported sizes ranging from 30-100 nm^[56]^ or provided an indicated value of 161.9 ± 54.8 nm^[57]^. EVs produced by *L. rhamnosus* JB-1 have a mean size of 130 nm and a mode value of 145 nm^[58]^. Therefore, the properties of vesicular structures characterized for the two strains in this study are consistent with the morphology and dimensions described for EVs produced by other representatives of this genus.

Proteomic analysis demonstrated that several proteins characterized in *L. plantarum* PCM 2675 EVs are membrane-located, similarly as described in the other proteomic studies of EVs derived from *L. plantarum* strains WCFS1, BGAN8, JCM8341, and BCRC 10069^[47-49,59]^. Herein, numerous proteins involved in carbohydrate metabolic pathways, cell division, transport, cell wall homeostasis and communication were found within the protein cargo of LpEVs. For EVs from *L. rhamnosus* PCM 489, identified proteins included cell metabolism enzymes, membrane proteins related to transport, and enzymes involved in cell wall remodeling and catabolism. In the identification of vesicular proteins for the *L. plantarum* strain BGAN8, numerous membrane proteins and transport-related proteins were also found as EVs cargo^[47,48]^. Some of the identified vesicular proteins, including glyceraldehyde-3-phosphate dehydrogenase (GAPDH), identified both in LpEVs and LrEVs, might be involved in the interactions with host proteins mucin and fibronectin^[60]^. GAPDH was also identified in EVs produced by *L. plantarum* in other studies^[48]^. Moreover, in LpEVs, additional mucus-binding proteins were identified herein, as well as D- and L-lactate dehydrogenases, enzymes crucial for bacterial physiology and interactions with host and other microorganisms that constitute microbiome^[61,62]^. Additionally, ABC transporters and bacteriocin transport accessory proteins that were identified in this work in bacterial EVs, are involved in the secretion of antimicrobial peptides - bacteriocins^[63]^, and the cysteine peptidases from the C39 family are responsible for hydrolysis of the leader peptides from the precursors of various bacteriocins^[64]^. In the proteomic studies of EVs from other strains and species of *Lactobacillaceae*, molecules such as lipoproteins, lactate dehydrogenase, mucus binding proteins, ABC transporters, peptidases and lysozyme were also identified^[48,49,50,59]^, which indicates important similarities in the protein content of EVs characterized for different representatives of lactobacilli. In addition, the presence of hydrolases among vesicular proteinaceous cargo and the hydrolytic activity presented by bacterial EVs might be related to the mechanism of their release from the bacterial cell, which is not yet sufficiently well understood for Gram-positive bacteria, but possibly can involve the degradation of cell wall peptidoglycan by enzymes, including lysozyme and peptidases, carried within or at the surface of membrane vesicles^[49,65]^. Several types of proteases have been identified so far within vesicles of other species of *Lactobacillaceae*, including zinc metalloproteases and serine proteases^[48,49,59]^. Importantly, this study demonstrates the genuine proteolytic activity of bacterial EVs, supported by the proteomic identification of several potential proteases within EVs cargo. As a wide range of proteases produced by lactic acid bacteria demonstrate specificity toward the milk protein casein^[66]^, this protein was also used herein to demonstrate that the proteases in EVs produced by the two bacterial strains tested are also hydrolytically active. The proteolytic activity of lactic acid bacteria is favorably related to their application in industry, i.e., in food fermentation, as their probiotic properties significantly increase the health benefits for consumers of such products^[67]^.

The involvement of EVs released by probiotic bacteria in the different types of interactions with host cells has been repeatedly demonstrated; thus, the verification of their non-toxicity for the host is of particular importance^[26]^. In our study, the cytotoxic effect of LpEVs and LrEVs was tested using the A549 epithelial cell line isolated from lung tissue of lung cancer patient^[68]^ and an *in vivo* invertebrate model of *G. mellonella* larvae. Epithelial cells are often the first line of contact with probiotics or postbiotics after their administration. The decision to use this specific cell line was based on reports indicating that oral or respiratory administration of probiotics, including *L. plantarum* and *L. rhamnosus*, or postbiotics derived therefrom, can be beneficial for host during different respiratory diseases, including bronchopulmonary dysplasia, chronic obstructive pulmonary disease, pneumonia, cystic fibrosis, infection with influenza virus, and lung cancer^[69,70]^. The changes observed in these studies were not statistically significant, but some very slight effects were detectable. In other studies, there were no significant changes in the metabolic activity of Caco-2 cells after 24-h incubation with EVs from *L. plantarum* WCFS1^[47]^. When the toxicity of EVs from *L. plantarum* BGAN8 was tested with the human colonic cell line HT29 monolayers, no significant change was noticed^[48]^. The treatment of murine macrophages of the RAW 264.7 cell line and intestinal epithelial cells of the MODE-K cell line with EVs produced by *L. murinus* demonstrated no decrease in the viability of host cells^[71]^. The EVs from *L. crispatus* strain RIGLD-1, used at concentrations ranging from 5 to 50 µg/mL, did not affect the metabolic activity of human gastric adenocarcinoma AGS cells^[72]^. However, for human liver cancer cell line HepG2, EVs from *L. rhamnosus* GG decreased the cell metabolic activity^[56]^, and for colorectal cancer cells from cell lines Sw480 and HT29, EVs from *L. rhamnosus* GG caused significant inhibition of metabolic activity^[73]^. Treatment of human dermal fibroblasts with EVs released by *L. paracasei* at various concentrations resulted in a noticeable decrease in the fibroblast viability at the highest concentration analyzed (100 µg/mL). In contrast, no decrease in viability was observed at lower concentrations ranging from 1 to 50 µg/mL^[74]^. Moreover, after stimulation of fibroblasts with TNF-α, the addition of *L. paracasei* EVs at low concentration resulted in a slight increase in cell viability^[74]^. The effects of EVs from *Saccharomyces boulardii* CNCM I-745 on the metabolic activity of three human intestinal cell lines were examined, involving the cancer cell lines HT-29 and HCT116, as well as the normal CCD841 CoN cell line^[75]^. The obtained results showed a slight decrease in metabolic activity at the highest concentration of *S. boulardii* EVs for both cancerous cell lines. In contrast, an increase in mitochondrial dehydrogenase activity was observed in the CCD841 CoN cells across all tested EVs concentrations; however, the effect was smallest at the highest EVs concentration^[75]^. In the research of Kim *et al*., EVs of *Bifidobacterium longum* KACC 91563 induced apoptosis in mast cells, while for T cells, B cells, and eosinophils, the induction of cell death was not detected^[76]^. Moreover, the observed effect in the induction of apoptosis of mast cells by EVs was more significant than by whole cells of *B. longum*^[76]^. For one of the symbiont bacteria-*Akkermansia muciniphila-*the co-incubation of bacterial EVs with Caco-2 cells for 24 h showed a significant decrease in the metabolic activity of host cells only at the highest concentration of EVs equal to 50 µg/mL. The other used concentrations of *A. muciniphila* vesicles did not induce significant changes in cell viability^[77]^.

In addition to *in vitro* tests, it is also important to analyze the impact of EVs on the host in the *in vivo* approach^[43]^. In such a model, the involvement of many different cell types and host systems in response to a stimulus is considered. Previously, we tested the mortality of *G. mellonella* larvae after injection of EVs derived from probiotic bacteria *S. salivarius* K12 and yeast *S. boulardii* CNCM I-745, and there was no statistically significant difference in larval survival between the EVs and the DPBS injection^[29]^. In the current study for EVs from *L. plantarum* and *L. rhamnosus* tested strains, the effect on larvae viability was also not statistically significant; however, a minor reduction in the survival rate was observed, which correlated with the increased amount of injected EVs.

The characterization of EVs from *L. plantarum* PCM 2675 and *L. rhamnosus* PCM 489 showed that these structures are similar to EVs previously described for other strains within the same species. Specifically, they are comparable in size, proteomic content, and effect on host cells. Therefore, the EVs isolation method employed in this work produced structures with features similar to those obtained in other studies, but with a shorter procedure time. In addition, the presented results provided evidence for the potential applications of LpEVs and LrEVs in different contexts where probiotic properties could benefit host health. However, careful observation of the effects of probiotic vesicular structures on the host during functional studies is certainly required, especially regarding the dependence of the response on the EVs concentration and method of administration.

A potential future application of probiotic-derived EVs may be their use as drug nanocarriers directed to a specific therapeutic target^[78,79]^. A significant advantage of EVs released by bacteria is their rapid production capacity, which surpasses that of mammalian systems. Moreover, probiotic EVs are well-tolerated by the host organism, as they originate from microorganisms that are part of the physiological microbiota^[45]^. A particularly promising strategy is the engineering of bacterial vesicles, based either on the genetic modification of selected strains to increase the production of EVs or to specifically change their properties and content, or direct modification of already produced bacterial structures^[80]^. Their application in treating various disorders, including chronic wounds, central nervous system diseases, cancer, and osteoporosis, is currently intensively studied^[80-82]^. An interesting example of such an approach was the construction of recombinant probiotic strain *Escherichia coli* Nissle 1917 with overexpression of chemokine receptor CXCR4 and growth factor BMP-2, which were subsequently presented on the surface of produced EVs as fusion proteins with bacterial surface protein ClyA^[83,84]^. Thus, engineered vesicles have been later employed in the approach to the treatment of osteoporosis in an ovariectomized mouse model, yielding promising results^[84]^. Research attempts are also in progress to enable various vesicular preparations to be administered not only intravenously, but also superficially, orally or through aerosols, which would significantly facilitate the therapeutic process and reduce the inconvenience for patients^[85,86]^. Considering the health benefits of probiotics and the versatile applications of modified EVs, enhancing the targeting and effectiveness of probiotic-derived EVs, as well as combining them with other materials, could significantly contribute to the design and development of innovative therapeutic strategies.

## DECLARATIONS

### Availability of data and materials

The datasets generated during the current study are available in the Cracow Open Research Data Repository, https://doi.org/10.57903/UJ/K93MBY. The mass spectrometry proteomics data have been deposited to the ProteomeXchange Consortium via the PRIDE partner repository^[32]^ with the dataset identifiers PXD048874 (*L. plantarum*) and PXD048876 (*L. rhamnosus*).

### Copyright

© The Author(s) 2024.
