# Supplementary material for: Extracellular vesicles of Lactiplantibacillus plantarum PCM 2675 and Lacticaseibacillus rhamnosus PCM 489: an introductory characteristic
Source: Extracell Vesicles Circ Nucl Acids. 2024 Nov 7;5(4):580–96. doi: 10.20517/evcna.2024.49 (PMC11725429; doi:10.20517/evcna.2024.49)
Supplement: Supplementary file 1 [file evcna-5-4-580-SupplementaryMaterials.pdf]

Supplementary Materials

Extracellular vesicles of *Lactiplantibacillus plantarum* PCM 2675 and *Lacticaseibacillus rhamnosus* PCM 489: an introductory characteristic

Katarzyna Kowalik[1,2], Kamila Kulig[1], Elzbieta Karnas[3], Olga Barczyk-Woznicka[4], Ewa Zuba-Surma[3], Elzbieta Pyza[4], Maria Rapala-Kozik[1], Justyna Karkowska-Kuleta[1]

[1]Department of Comparative Biochemistry and Bioanalytics, Faculty of Biochemistry, Biophysics and Biotechnology, Jagiellonian University, Kraków 30-387, Poland.
[2]Doctoral School of Exact and Natural Sciences, Faculty of Biochemistry, Biophysics and Biotechnology, Jagiellonian University, Kraków 30-387, Poland.
[3]Department of Cell Biology, Faculty of Biochemistry, Biophysics and Biotechnology, Jagiellonian University, Kraków 30-387, Poland.
[4]Department of Cell Biology and Imaging, Institute of Zoology and Biomedical Research, Jagiellonian University, Kraków 30-387, Poland.

Correspondence to: Dr. Justyna Karkowska-Kuleta, Department of Comparative Biochemistry and Bioanalytics, Faculty of Biochemistry, Biophysics and Biotechnology, Jagiellonian University, Gronostajowa 7, Kraków 30-387, Poland. E-mail: justyna.karkowska@uj.edu.pl

Table 1. Mass spectrometry identification of proteins from *Lactiplantibacillus plantarum* PCM 2675 extracellular vesicles (EVs).

Accession Number	Protein Description	Score	Sequence Coverage [%]	Molecular Mass [kDa]	pI
**A0A9X4R8V7**	SH3 domain-containing protein	2418.94	61.33	81.7	8.92
**A0A6P1PM54**	Mucus-binding protein	1505.36	34.34	236.1	4.86
**F9UP14**	Mucus-binding protein. LPXTG-motif cell wall anchor	1390.75	31.10	236.0	4.86
**A0A166PAQ3**	Gamma-D-glutamyl-meso-diaminopimelate peptidase	1097.12	27.64	34.7	9.39
**A0A165RVQ5**	Lysozyme	989.42	9.51	83.2	8.06
**F9UM10**	Glyceraldehyde-3-phosphate dehydrogenase	676.53	39.71	36.4	5.54
**A0A0G9F8U8**	Elongation factor Tu	619.61	46.58	43.4	5.08
**A0A6P1PJE6**	ESX secretion system protein YueB	594.97	17.84	127.9	9.03
**A0A1E3KUY5**	N-acetylmuramoyl-L-alanine amidase sle1	499.47	33.66	21.1	8.91
**A0A1W6NN01**	Transglycosylase	484.98	25.98	25.2	8.97
**A0A8I1ZFG3**	LPXTG cell wall anchor domain-containing protein	446.97	17.99	86.1	8.34
**Q88VJ2**	D-lactate dehydrogenase	412.30	50.60	37.2	5.06
**A0A0G9F7Y4**	Extracellular protein	384.46	33.48	23.2	9.89
**A0A387DSN5**	Penicillin-binding protein 1A	365.88	18.22	82.6	9.42
**A0A0G9F7S8**	FAD-dependent oxidoreductase	349.46	38.58	48.2	5.68
**A0A0G9FB10**	DNA-binding protein HU	311.53	76.92	9.6	9.76
**A0A809NVH3**	LysM domain protein	304.84	16.15	31.9	9.55
**A0A166AYS7**	Extracellular zinc metalloproteinase	293.55	28.37	23.9	8.50
**A0A410LXP1**	Putative peptidoglycan endopeptidase LytE	277.44	14.52	48.3	9.09
**A0A0G9FDU4**	Class II fructose-1.6-bisphosphate aldolase	272.59	33.10	30.9	5.20
**K4EKH6**	Extracellular protein D1 (Fragment)	259.99	33.70	19.4	7.49
**A0A1W6NUJ8**	Triosephosphate isomerase	239.83	37.76	25.7	4.64
**A0A0R2GA25**	Lysin	233.39	20.05	48.4	9.63
**A0A2S1T6M3**	L.D-transpeptidase catalytic domain-containing protein	232.63	18.58	36.6	10.27
**A0A0R2GDX5**	Acyltransferase	221.10	12.98	72.8	9.86
**A0A0G9GNN1**	Peptidase Do	215.54	14.05	43.1	9.55
**Q88X05**	Foldase protein PrsA 1	206.11	20.13	32.6	9.77
**A0A151G502**	Extracellular protein	200.18	17.52	29.3	10.62
**A0A1E3KQK8**	15 kDa lipoprotein	194.05	27.45	32.8	9.76
**A0A0M4D5D3**	Extracellular protein	191.98	11.18	34.2	9.51
**A0A2S1T6U3**	NAD(P)/FAD-dependent oxidoreductase	191.30	16.36	47.6	5.36
**A0A151G276**	Glycoprotein gp2	187.81	23.29	30.9	9.66
**A0A165BUG4**	DNA helicase	187.29	14.02	34.3	6.00
**A0A0L7Y402**	Co-chaperonin GroES	176.41	50.00	10.3	5.03
**A0A0G9FCV9**	Acid shock protein	174.00	28.57	16.0	4.70
**A0A0R2GEM1**	L-lactate dehydrogenase	168.80	16.09	33.9	5.05
**A0A0G9FA95**	Cell envelope-associated transcriptionalattenuator LytR-CpsA-Psr subfamily F2	161.65	18.55	37.7	9.89
**A0A1E3KRD4**	Phosphoglycerate kinase	161.12	15.75	42.8	5.25
**A0A0M5INP1**	Amino acid ABC transporter substrate-binding protein	159.38	19.10	28.9	9.96
**A0A151G3U8**	Glutathione reductase	156.76	17.27	47.6	5.53
**A0A0G9FH93**	Adherence protein. chitin-binding domain protein	153.66	25.87	22.2	8.85
**A0A151G8Y6**	Fructose permease IID component	143.78	17.10	34.3	9.39

**A0A0G9F8I8**	Chaperone protein DnaK	142.70	6.11	66.7	4.84
**A0A0R2G3W5**	Chaperonin GroEL	137.17	12.57	57.4	4.81
**A0A165P2U7**	Enolase	131.32	7.69	48.0	4.74
**A0A165C255**	PTS beta-glucoside transporter subunit IIABC	130.67	5.11	69.4	8.47
**A0A0G9FCB2**	Large ribosomal subunit protein uL11	130.02	35.46	14.8	9.23
**A0A162GIN5**	Extracellular protein	126.56	6.45	22.7	9.11
**A0A0G9F8J5**	Large ribosomal subunit protein bL12	125.48	53.28	12.6	4.48
**A0A151G3H2**	Capsid protein	123.53	17.56	45.4	5.11
**A0A0R2GCN4**	Extracellular zinc metalloproteinase	119.87	11.11	27.5	8.13
**A0A0R2G594**	Acyltransferase	117.49	11.48	66.9	9.09
**A0A0G9FF02**	Cell surface protein	116.85	10.36	23.1	10.59
**A4F3M9**	ATP synthase alpha subunit (Fragment)	112.29	18.28	30.1	5.40
**A0A0R2GKD9**	D-alanyl-D-alanine carboxypeptidase	107.67	3.16	45.2	9.79
**A0A0G9FJ83**	ABC-type oligopeptide transport system. periplasmic component	101.26	7.50	60.3	9.64
**A0A0M3QB24**	Dipeptidase PepV	96.12	6.00	50.8	4.65
**A0A0R2G5Y4**	inorganic diphosphatase	90.43	23.62	33.6	4.69
**A0A151G9J5**	DUF5067 domain-containing protein	90.11	19.35	20.5	8.79
**A0A0G9FDS9**	Large ribosomal subunit protein bL21	89.87	31.37	11.0	9.55
**A0A0G9GTF0**	Preprotein translocase YajC	88.99	10.24	13.8	4.94
**A0A166J7K5**	DUF4115 domain-containing protein	87.87	4.48	31.3	9.44
**A0A0G9FAE9**	FAD:protein FMN transferase	84.36	9.19	40.2	9.73
**A0A0G9FA51**	ATP synthase subunit beta	83.04	6.00	50.8	4.72
**A0A0G9FEF6**	2.5-diketo-D-gluconic acid reductase	80.65	20.63	31.7	5.67
**A0A0G9FEW8**	Maltodextrin-binding protein	80.53	9.11	45.6	9.69
**A0A7Z2PAN8**	Holin	72.89	10.48	12.7	4.72
**A0A0G9GUZ2**	Small ribosomal subunit protein uS11	70.68	10.08	13.8	11.68
**A0A809NRY6**	Cell surface protein. ErfK family	68.72	10.32	14.1	10.58
**A0A151G810**	Foldase protein PrsA	66.87	7.12	34.3	9.94
**A0A0L7Y4S1**	Alkaline shock protein 23	65.95	27.86	15.8	5.06
**A0A1E3KTH1**	Universal stress protein	65.14	10.63	17.5	6.33
**A0A0G9FH87**	DUF805 domain-containing protein	63.11	11.57	14.2	8.56
**A0A0G9FFK7**	Signal peptidase I	62.56	12.82	22.4	9.89
**A0A0G9F9A6**	Glutamine transport system permease proteinGlnP	60.64	2.93	52.2	9.64
**A0A6P1PFD4**	Large ribosomal subunit protein uL29	59.54	20.31	7.6	9.92
**A0A0G9F817**	ATP synthase subunit b	58.87	6.43	18.7	5.96
**A0A165MMQ4**	YtxH domain-containing protein	58.02	8.33	15.4	4.51
**A0A0G9GW61**	CsbD family protein	57.79	12.33	8.0	9.14
**A0A0R2G641**	DNA-directed RNA polymerase subunit beta'	56.18	0.91	134.2	7.17
**A0A0G9F8V1**	Pyruvate kinase	55.84	5.46	62.8	5.14
**A0A0M0CDK3**	ABC amino acid transpor. substrate binding protein	55.70	5.40	29.9	9.74
**A0A0M4CRF8**	Manganese transport protein MntH	55.18	5.06	51.2	8.62
**A0A0G9FCE5**	Lipoprotein	54.95	5.73	17.3	10.27
**A0A0G9F8M5**	PTS system mannose-specific EIIAB component	54.84	2.47	35.3	6.38
**A0A0R2G7R4**	Lipoprotein	54.37	4.21	41.3	9.01
**A0A0R2G0X6**	Glucose-6-phosphate isomerase	53.76	16.44	49.8	5.03
**A0A0G9GTR0**	Major capsid protein	52.72	9.12	37.6	5.11
**A0A0R2GCV0**	Serine-rich adhesin for platelets	51.56	3.12	74.9	9.57

**A0A2S1TDB8**	L.D-transpeptidase YciB	50.09	8.78	23.7	9.32
**A0A0M0CJM6**	Phosphoenolpyruvate-protein phosphotransferase	49.84	2.08	63.1	4.83
**A0A809PTB3**	Fusion of IIA. IIB and IIC component of mannitol/fructose-specific phosphotransferase system mannitol/fructose-specific	49.35	2.18	67.0	5.90
**A0A162EDM2**	18 kDa heat shock protein	49.12	12.24	16.6	5.25
**A0A809NSF1**	DNA-entry nuclease	46.97	7.67	31.7	10.36
**A0A1W6NR97**	Chaperone protein ClpB	46.79	2.67	95.9	5.31
**A0A0G9F9R6**	Large ribosomal subunit protein uL16	45.89	9.72	16.1	10.46
**A0A151G0Q2**	DNA starvation/stationary phase protection protein (Fragment)	45.27	19.12	15.8	4.94
**A0A0G9FHI2**	Large ribosomal subunit protein bL27	45.15	13.04	10.1	10.95
**A0A836NNX2**	Uncharacterized protein	43.38	4.67	23.7	9.33
**A0A0G9GQ85**	LapA family protein	41.90	21.55	12.7	10.08
**A0A0G9FBB9**	Uncharacterized protein	41.63	14.16	13.0	4.65
**A0A0L7Y0E2**	Elongation factor Ts	41.52	3.08	31.6	5.01
**A0A151G9H1**	Peptide ABC transporter substrate-binding protein	38.40	3.70	59.9	9.61
**A0A151G4G0**	Endolytic murein transglycosylase	38.12	3.08	39.6	9.57
**A0A1E3KSG8**	phosphopyruvate hydratase	37.67	7.41	8.9	7.43
**A0A0L7Y2W3**	CNT family concentrative nucleoside transporter	37.63	6.56	46.3	5.52
**A0A0M4CU25**	Cytochrome D Ubiquinol oxidase subunit I	36.05	1.88	53.7	9.17
**Q88V33**	Probable transcriptional regulatory protein lp_2253	35.54	5.37	26.4	4.87
**A0A1E3KNC6**	Putative membrane protein YdgH	35.36	0.99	137.8	9.54
**A0A0R2GCG2**	Peptidase C39-like domain-containing protein	35.18	9.24	26.2	8.07
**A0A0G9F7C8**	Transcription termination factor Rho	34.67	1.86	48.9	9.52
**A0A0R2GHJ7**	Putative ErfK/YbiS/YcfS/YnhG family protein	34.67	2.63	50.3	9.52
**A0A0M0CEL8**	CDP-diacylglycerol--glycerol-3-phosphate 3-phosphatidyltransferase	34.48	5.15	21.4	9.17
**A0A0G9FBH5**	Membrane protein	34.28	5.19	24.1	9.70
**M9R0M5**	DNA-directed RNA polymerase (Fragment)	34.18	12.22	10.1	11.37
**A0A7Z3KWS6**	Bacteriocin transport accessory protein	34.03	3.93	20.3	9.72
**A0A0G9FCP9**	LemA family protein	31.76	6.99	20.8	6.95
**A0A0G9F6G3**	Guanosine 5-monophosphate oxidoreductase	31.62	3.13	39.9	5.78
**A0A0G9F9W6**	UvrABC system protein B	30.42	1.65	76.1	5.20
**A0A0G9F9U0**	Large ribosomal subunit protein uL6	30.38	3.93	19.3	9.55
**A0A0M4RSV1**	serine-type D-Ala-D-Ala carboxypeptidase	29.75	2.25	46.9	9.89
**A0A1E3KSZ9**	Integral membrane protein	28.86	1.33	60.0	10.04
**A0A0G9FB84**	Cell cycle protein GpsB	27.86	15.04	12.9	6.57
**A0A0R2G6L9**	Amino acid ABC transporter ATP-binding protein	27.58	4.10	27.2	5.71
**A0A0G9F9U2**	Phosphate-binding protein	27.12	5.84	31.5	9.82
**A0A0G9FIW0**	Glucosamine-6-phosphate deaminase	26.56	6.75	25.9	5.08
**A0A8I1ZL21**	Aspartate-alanine antiporter	26.10	2.36	59.1	8.12
**A0A0G9GPI1**	ABC polar amino acid transport system. ATPase component	25.95	4.51	26.8	5.48
**A0A0G9FBB8**	Ribosome hibernation promoting factor	24.42	13.83	21.7	5.54
**A0A2S1T8N9**	Protein GrpE	23.83	5.53	21.4	5.01
**A0A0G9F9S5**	Large ribosomal subunit protein uL15	23.51	7.69	15.3	10.64
**A0A807DKI6**	Cell surface protein	23.51	2.79	38.0	9.17
**A0A347ZFY2**	Short-chain dehydrogenase	23.10	2.83	26.5	6.83
**A0A0G9GK27**	Ribosome-recycling factor	20.09	9.63	20.6	6.11
**A0A166MST0**	Large ribosomal subunit protein uL18	20.00	61.33	81.7	8.92

Accession Number	Protein Description	Score	Sequence Coverage [%]	Molecular Mass [kDa]	pI
**A0A9X4R8V7**	SH3 domain-containing protein	3676.83	66.20	81.7	8.92
**A0A166PAQ3**	Gamma-D-glutamyl-meso-diaminopimelate peptidase	2732.75	31.62	34.7	9.39
**A0A6P1PM54**	Mucus-binding protein	2419.14	35.96	236.1	4.86
**A0A809PD29**	Cell surface protein	2142.47	31.12	236.0	4.88
**F9UP14**	Mucus-binding protein. LPXTG-motif cell wall anchor	2140.36	33.53	236.0	4.86
**A0A809NVY5**	Cell wall hydrolase/muramidase	1984.21	56.99	78.8	8.66
**A0A165RVQ5**	Lysozyme	1327.17	12.15	83.2	8.06
**A0A836ZGR3**	Cell surface protein	1209.10	36.93	86.1	8.72
**F9UM10**	Glyceraldehyde-3-phosphate dehydrogenase	1161.53	65.00	36.4	5.54
**A0A387DSN5**	Penicillin-binding protein 1A	1158.07	39.45	82.6	9.42
**A0A8I1ZFG3**	LPXTG cell wall anchor domain-containing protein	1117.42	31.53	86.1	8.34
**A0A6P1PJE6**	ESX secretion system protein YueB	1034.99	25.22	127.9	9.03
**A0A809NVH3**	LysM domain protein	1002.19	29.19	31.9	9.55
**A0A1W6NN01**	Transglycosylase	954.99	27.17	25.2	8.97
**A0A1E3KUY5**	N-acetylmuramoyl-L-alanine amidase sle1	886.02	33.66	21.1	8.91
**Q88X05**	Foldase protein PrsA 1	810.37	37.25	32.6	9.77
**A0A1E3KQK8**	15 kDa lipoprotein	808.49	38.56	32.8	9.76
**A0A0R2G3W5**	Chaperonin GroEL	766.28	43.44	57.4	4.81
**A0A165BUG4**	DNA helicase	740.81	42.68	34.3	6.00
**A0A0G9FB10**	DNA-binding protein HU	716.06	83.52	9.6	9.76
**A0A0G9F7Y4**	Extracellular protein	710.42	51.13	23.2	9.89
**A0A410LXP1**	Putative peptidoglycan endopeptidase LytE	702.17	23.19	48.3	9.09
**A0A0G9FJ83**	ABC-type oligopeptide transport system. periplasmic component	603.70	30.16	60.3	9.64
**A0A0G9FEW8**	Maltodextrin-binding protein	598.17	34.77	45.6	9.69
**A0A0G9FCV9**	Acid shock protein	587.92	80.71	16.0	4.70
**A0A387DN00**	Lipoprotein	581.01	52.38	31.0	9.67
**A0A0L7Y402**	Co-chaperonin GroES	574.56	87.23	10.3	5.03
**Q5NJY8**	Triosephosphate isomerase	521.98	60.32	27.0	4.74
**A0A2S1T6M3**	L.D-transpeptidase catalytic domain-containing protein	521.37	18.27	36.6	10.27
**A0A0R2GA25**	Lysin	508.38	31.11	48.4	9.63
**A0A151G502**	Extracellular protein	492.65	42.70	29.3	10.62
**A0A151G2E0**	Serine-rich adhesin for platelets	483.62	12.78	75.8	9.63
**Q88VJ2**	D-lactate dehydrogenase	462.39	40.66	37.2	5.06
**K4EKH6**	Extracellular protein D1 (Fragment)	450.85	45.65	19.4	7.49
**A0A0G9GNN1**	Peptidase Do	443.87	38.10	43.1	9.55
**A0A166AYS7**	Extracellular zinc metalloproteinase	437.27	52.88	23.9	8.50
**A0A0G9FA95**	Cell envelope-associated transcriptionalattenuator LytR-CpsA-Psr subfamily F2	429.51	48.41	37.7	9.89
**A0A1A0DI94**	Foldase protein PrsA	388.84	44.98	34.3	9.98
**A0A151G9H1**	Peptide ABC transporter substrate-binding protein	387.41	25.56	59.9	9.61
**Q5NJY9**	Phosphoglycerate kinase	385.25	49.25	42.8	5.20
**A0A0G9FDU4**	Class II fructose-1.6-bisphosphate aldolase	369.03	29.97	30.9	5.20
**A0A0R2GDX5**	Acyltransferase	353.77	12.67	72.8	9.86
**A0A0M4D5D3**	Extracellular protein	351.12	23.60	34.2	9.51

**A0A0M4RSV1**	Serine-type D-Ala-D-Ala carboxypeptidase	350.33	26.13	46.9	9.89
**A0A0G9F834**	Lipoprotein	345.25	32.85	29.9	9.85
**A0A0G9FH93**	Adherence protein. chitin-binding domain protein	336.92	32.34	22.2	8.85
**A0A0R2GEM1**	L-lactate dehydrogenase	332.84	20.19	33.9	5.05
**A0A0M5INP1**	Amino acid ABC transporter substrate-binding protein	314.32	40.45	28.9	9.96
**A0A151G9J5**	DUF5067 domain-containing protein	313.30	37.10	20.5	8.79
**A0A0P7JVD2**	Beta-lactamase family protein	296.21	21.23	43.3	9.80
**A0A0G9F8U8**	Elongation factor Tu	294.69	32.15	43.4	5.08
**A0A0G9FEF6**	2.5-diketo-D-gluconic acid reductase	290.69	41.61	31.7	5.67
**A0A165P2U7**	Enolase	289.43	25.34	48.0	4.74
**A0A151G3H2**	Capsid protein	282.06	23.41	45.4	5.11
**A0A0R2G5Y4**	Inorganic diphosphatase	281.37	29.45	33.6	4.69
**A0A9X4R928**	Ig-like domain-containing protein	275.67	17.83	60.5	4.48
**A0A165P4L0**	Prophage Lp1 protein 66 lipoprotein	269.31	26.58	23.1	10.59
**A0A0G9FAH1**	Extracellular transglycosylase. membrane-bound	262.40	25.95	15.0	10.48
**A0A387DZJ7**	YtxH domain-containing protein	253.66	44.67	16.0	4.54
**A0A151G3U8**	Glutathione reductase	251.18	22.95	47.6	5.53
**A0A0G9F7S8**	FAD-dependent oxidoreductase	243.29	26.39	48.2	5.68
**A0A0G9FAE9**	FAD:protein FMN transferase	229.76	21.35	40.2	9.73
**A0A0G9F8J5**	Large ribosomal subunit protein bL12	223.06	61.48	12.6	4.48
**A0A0G9GK27**	Ribosome-recycling factor	211.65	43.32	20.6	6.11
**A0A0M0CDK3**	ABC amino acid transport. substrate binding protein	208.80	22.30	29.9	9.74
**A0A0G9F8I8**	Chaperone protein DnaK	206.74	22.35	66.7	4.84
**A0A0R2GDU4**	Penicillin-binding protein	202.48	12.68	72.7	9.41
**A0A347ZH70**	ATP synthase subunit b	201.06	30.41	18.7	5.96
**A0A0L7Y3C4**	DNA protection during starvation protein	180.72	72.26	18.0	4.79
**A0A165S6Z5**	Extracellular protein gamma-D-glutamate-meso-diaminopimelate muropeptidase	178.03	19.75	27.0	10.35
**A0A0G9FCB2**	Large ribosomal subunit protein uL11	173.93	38.30	14.8	9.23
**A0A1A0DFB7**	Cell surface protein. CscB family	172.60	19.26	24.5	4.83
**A0A9X4LRC1**	LysM peptidoglycan-binding domain-containing protein	171.03	22.61	30.7	9.14
**A0A0R2G7R4**	Lipoprotein	168.58	15.79	41.3	9.01
**A0A165P7L5**	DNA-entry nuclease	167.70	18.97	31.8	9.72
**A0A1E3KTH1**	Universal stress protein	165.69	25.62	17.5	6.33
**A0A0R2GCN4**	Extracellular zinc metalloproteinase	165.08	29.89	27.5	8.13
**A0A0M3QB24**	Dipeptidase PepV	164.97	14.56	50.8	4.65
**A0A162EDM2**	18 kDa heat shock protein	164.84	46.26	16.6	5.25
**A0A6P1PFD4**	Large ribosomal subunit protein uL29	161.79	70.31	7.6	9.92
**A0A0G9FDS9**	Large ribosomal subunit protein bL21	155.50	33.33	11.0	9.55
**A0A0G9F8L8**	Altered inheritance of mitochondria protein 41. mitochondrial	154.78	53.74	16.1	5.39
**A0A0L7Y4S1**	Alkaline shock protein 23	154.56	27.86	15.8	5.06
**A0A1E3KNC6**	Putative membrane protein YdgH	153.71	4.71	137.8	9.54
**A0A809NTI9**	ABC transporter substrate-binding protein	150.25	18.1	33.1	9.60
**A0A0R2G0X6**	Glucose-6-phosphate isomerase	148.34	9.56	49.8	5.03
**A0A162GIN5**	Extracellular protein	147.86	7.37	22.7	9.11
**A0A0R2G594**	Acyltransferase	147.29	14.14	66.9	9.09
**A0A0R2GCG2**	Peptidase C39-like domain-containing protein	145.10	13.87	26.2	8.07

**A0A162FHL3**	Gram-positive cocci surface proteins LPxTG domain-containing protein	143.13	7.61	93.5	5.83
**A0A0G9F9R6**	Large ribosomal subunit protein uL16	142.48	27.08	16.1	10.46
**A0A0G9FBV7**	Large ribosomal subunit protein uL24	139.88	45.63	11.4	9.99
**A0A7Z2PAB4**	Extracellular protein	138.28	25.43	18.5	8.24
**A0A0G9GQI8**	Alpha/beta hydrolase	134.98	23.42	29.6	9.04
**A0A0G9FAW9**	PTS sugar transporter subunit IIB	132.11	51.43	11.3	4.75
**A0A0G9GUZ2**	Small ribosomal subunit protein uS11	123.79	12.40	13.8	11.68
**A0A0M0CJM6**	Phosphoenolpyruvate-protein phosphotransferase	120.53	9.55	63.1	4.83
**A0A162G2N8**	Sortase A LPXTG specific	119.95	15.45	25.8	10.02
**A0A2S1T8N9**	Protein GrpE	116.71	21.11	21.4	5.01
**A0A162E5U7**	Uncharacterized protein	115.86	27.27	4.6	8.92
**A0A0G9FE27**	Transcription elongation factor GreA	115.79	21.88	17.9	4.67
**A0A0G9F9U0**	Large ribosomal subunit protein uL6	113.79	24.72	19.3	9.55
**A0A6P1PHJ1**	SDR-like Ig domain-containing protein	112.35	10.46	42.7	4.87
**A0A0G9FAC7**	ABC transporter permease	112.32	7.33	53.8	9.82
**A0A0G9F9R0**	Large ribosomal subunit protein uL23	109.91	31.96	11.1	9.66
**A0A0L7Y2A2**	Aldo/keto reductase	108.57	18.62	31.9	5.25
**A0A0G9F766**	Cell shape-determining protein MreC	107.46	11.03	30.1	9.76
**A0A0R2GGQ6**	LytR family transcriptional regulator	105.60	9.04	37.7	9.73
**A0A0G9FAD3**	Small ribosomal subunit protein uS5	102.89	21.69	17.3	9.64
**A0A0S2Z2N2**	Mucin-binding protein (Fragment)	101.71	6.71	49.7	4.91
**A0A0G9F979**	UPF0342 protein AVR83_14860	100.75	23.68	13.3	4.70
**A0A0G9GUN2**	Extracellular protein membrane-anchored	99.31	29.46	12.5	10.77
**A0A0G9F9S9**	Large ribosomal subunit protein uL2	99.16	8.60	30.2	10.55
**F2Y394**	S-ribosylhomocysteine lyase	99.12	19.62	17.4	6.52
**A0A0G9FBB9**	Uncharacterized protein	97.28	30.97	13.0	4.65
**A0A0G9FBU1**	Small ribosomal subunit protein uS12	95.39	22.63	15.2	11.25
**A0A0G9GS34**	Nucleotide-binding protein. universal stress protein UspA family	93.28	14.10	17.1	4.84
**A0A0L7Y0E2**	Elongation factor Ts	92.26	15.75	31.6	5.01
**A0A0G9FBE4**	Enoyl-[acyl-carrier-protein] reductase [NADH]	91.21	11.51	26.9	6.93
**A0A0G9FB84**	Cell cycle protein GpsB	90.27	42.48	12.9	6.57
**A0A807DKI6**	Cell surface protein	89.89	15.60	38.0	9.17
**A0A0G9FCE5**	Lipoprotein	88.52	18.47	17.3	10.27
**A0A1W6NP87**	Mucus-binding protein	87.90	2.08	135.2	5.01
**A0A0G9F8M5**	PTS system mannose-specific EIIAB component	86.50	10.80	35.3	6.38
**A0A0G9FAN5**	Small ribosomal subunit protein bS21	85.91	45.16	7.6	11.28
**A0A165R4V8**	Protein DltD	85.63	12.47	48.6	9.73
**A0A2S1TDB8**	L.D-transpeptidase YciB	85.41	21. 46	23.7	9.32
**A0A0R2GHJ7**	Putative ErfK/YbiS/YcfS/YnhG family protein	84.92	5.47	50.3	9.52
**A0A0G9GVC4**	Large ribosomal subunit protein bL20	84.51	25.42	13.6	11.34
**A0A0G9F9S5**	Large ribosomal subunit protein uL15	82.33	16.08	15.3	10.64
**A0A0G9FFR9**	Small ribosomal subunit protein uS19	81.41	21.98	10.3	9.82
**A0A165P7H9**	Integral membrane protein	76.42	3.61	66.7	9.48
**A0A0G9FHI2**	Large ribosomal subunit protein bL27	75.96	36.96	10.1	10.95
**A0A0G9FFK7**	Signal peptidase I	75.44	10.77	22.4	9.89
**A0A166J7K5**	DUF4115 domain-containing protein	75.01	4.48	31.3	9.44
**A0A0R2GKD9**	D-alanyl-D-alanine carboxypeptidase	73.76	9.95	45.2	9.79

**A0A0R2GDJ8**	Serine-type D-Ala-D-Ala carboxypeptidase	72.93	8.57	26.9	9.48
**A0A807DWA9**	Stress response regulator Gls24	72.56	9.44	19.5	5.14
**A0A0R2G2S8**	Signal peptidase I	72.26	7.58	22.5	10.08
**A0A7Z2SG82**	Ig-like domain-containing protein	71.79	1.32	279.2	4.81
**A0A7Z2PAN8**	Holin	70.45	10.48	12.7	4.72
**A0A0G9FCU1**	Peptidyl-prolyl cis-trans isomerase	69.14	5.67	21.1	4.68
**A0A0G9FAB6**	Large ribosomal subunit protein uL3	68.97	15.31	22.7	9.92
**A0A0R2G5K7**	Large ribosomal subunit protein uL13	68.85	15.49	15.5	9.52
**A0A0G9FE59**	Small ribosomal subunit protein bS20	68.38	34.52	9.1	10.52
**A0A0L7Y017**	Cell division protein FtsI (Peptidoglycansynthetase)	68.26	3.37	77.2	9.85
**A0A0G9GHJ1**	Large ribosomal subunit protein uL10	67.33	17.96	17.9	5.15
**A0A0P7J8K4**	Bis(5'-nucleosyl)-tetraphosphatase [asymmetrical]	66.71	10.07	15.7	4.77
**A0A0G9FAC1**	Large ribosomal subunit protein uL22	66.42	10.43	12.5	10.46
**A0A0M4RTT5**	Aldehyde/histidinol dehydrogenase	65.26	7.02	50.4	4.92
**A0A151G936**	DNA-entry nuclease	64.55	7.08	36.2	10.18
**A0A0G9FA91**	Uncharacterized protein	64.17	16.67	13.0	4.54
**A0A0G9GTC0**	Large ribosomal subunit protein bL31B	64.11	12.35	9.1	7.50
**A0A0R2GC63**	Small ribosomal subunit protein bS16	63.70	41.98	9.1	9.52
**A0A162EJQ0**	Uncharacterized protein	63.66	31.91	5.3	9.58
**A0A0G9FBU7**	Large ribosomal subunit protein uL4	63.36	15.46	22.6	10.11
**A0A0M0CJT7**	Cysteine desulfurase	63.28	18.58	12.6	8.94
**A0A9X4LR82**	Uncharacterized protein	62.35	30.77	5.6	11.75
**A0A0G9FFK6**	Alkaline shock protein 23	62.08	26.03	16.1	4.94
**A0A166MST0**	Large ribosomal subunit protein uL18	61.39	42.37	13.0	10.10
**A0A151GA06**	Prophage Lp2 protein 7	60.95	13.51	12.0	9.64
**Q88UU1**	ATP synthase subunit alpha	60.86	6.94	54.5	5.10
**A0A0G9FCP4**	Cell surface protein	60.79	11.48	23.5	9.79
**A0A0G9F715**	Ribokinase	60.57	3.27	31.7	5.30
**A0A0G9FBF0**	3-oxoacyl-[acyl-carrier-protein] synthase 2	59.55	7.32	42.4	5.39
**A0A0R2GG09**	Extracellular protein. peptide binding protein OppA-like protein	58.93	2.38	59.8	9.66
**A0A166KHH0**	phosphate acetyltransferase	58.23	3.08	34.4	5.17
**A0A0G9F9Q5**	Small ribosomal subunit protein uS7	58.13	8.97	17.8	9.82
**A0A0R2G6L9**	Amino acid ABC transporter ATP-binding protein	57.08	9.02	27.2	5.71
**A0A809NWV4**	Manganese/zinc ABC transporter. substrate binding protein	56.46	14.33	33.3	9.54
**A0A809NQT1**	Extracellular protein	56.08	8.04	53.8	9.64
**A0A1W6NUY7**	Metal ABC transporter substrate-binding protein	55.98	13.27	32.5	9.64
**A0A0G9F7G6**	Uncharacterized protein	55.66	7.65	19.4	9.67
**Q8KJS2**	Cupin domain-containing protein	55.29	14.77	16.4	5.69
**A0A0G9FBH0**	Cof-type HAD-IIB family hydrolase	55.27	4.50	31.3	5.11
**A0A1W6NUM2**	Sulfurtransferase	52.54	27.13	15.1	9.76
**A0A0M5IVC0**	Uncharacterized protein	51.80	43.14	5.9	11.09
**Q88V33**	Probable transcriptional regulatory protein lp_2253	51.36	5.37	26.4	4.87
**A0A0G9F850**	TPR repeat-containing thioredoxin TDX	51.12	10.38	12.3	4.70
**A0A151G265**	Ribitol-5-phosphate cytidylyltransferase	51.09	4.29	25.9	5.21
**Q88WU7**	Large ribosomal subunit protein bL35	51.02	42.19	7.4	12.04
**A0A0G9GVG8**	Nitroreductase	50.99	12.37	20.1	5.27
**A0A0G9F8V1**	Pyruvate kinase	50.80	2.22	62.8	5.14

**A0A0G9GQ85**	LapA family protein	49.84	22.41	12.7	10.08
**A0A0G9FAC2**	Trigger factor	49.59	5.91	49.4	4.63
**A0A809PTB3**	Fusion of IIA. IIB and IIC component of mannitol/fructose-specific phosphotransferase system mannitol/fructose-specific	48.02	2.18	67.0	5.90
**A0A0M3QAV6**	Aminopeptidase	47.35	1.30	93.9	4.88
**A0A0G9FFT5**	Large ribosomal subunit protein bL17	47.31	20.47	14.2	9.98
**A0A0G9F8V2**	Fe-S cluster biosynthesis protein	46.63	8.15	14.5	4.97
**A0A1E3KMX8**	Histone H1	45.83	7.41	28.7	10.98
**A0A0G9FBD6**	Thioredoxin reductase	45.64	3.85	33.4	5.01
**A0A0G9GS07**	Small ribosomal subunit protein uS14	45.59	21.31	7.2	10.59
**A0A0G9FBH5**	Membrane protein	45.08	5.19	24.1	9.70
**A0A151G7F1**	ACP phosphodiesterase	44.87	9.89	19.6	5.52
**A0A0G9FEG9**	NAD-dependent dehydratase	44.84	18.87	23.5	5.43
**A0A151G8Y6**	Fructose permease IID component	44.47	8.39	34.3	9.39
**A0A0G9FCP9**	LemA family protein	43.12	11.29	20.8	6.95
**A0A0R2G3D2**	Cell surface protein	42.99	4.96	38.1	10.01
**A0A0G9F931**	ribonucleoside-diphosphate reductase	42.71	2.68	39.1	4.61
**A0A0R2G4X3**	ATP-dependent Clp protease	42.41	1.45	76.1	5.77
**A0A809NUC1**	Metallophosphoesterase. lipoprotein	41.37	6.31	45.2	9.69
**A0A0G9FCV7**	Cell surface protein	40.49	11.05	20.1	4.79
**A0A0L7Y0X2**	Lipid II flippase MurJ	39.97	3.24	61.4	9.66
**A0A1E3KSZ9**	Integral membrane protein	39.46	1.33	60.0	10.04
**A0A0G9GPN4**	Diguanylate cyclase	38.68	7.37	20.4	5.17
**A0A0G9FBM2**	Large ribosomal subunit protein bL28	38.55	11.48	7.0	11.91
**A0A0G9F8J6**	DUF3892 domain-containing protein	37.65	12.50	11.0	5.48
**A0A0G9FFB0**	CdaA regulatory protein CdaR	37.57	8.31	34.6	9.52
**A0A166JKT2**	DUF1398 domain-containing protein	37.05	17.32	13.8	5.27
**Q88XW3**	Large ribosomal subunit protein bL36	36.71	25.64	4.6	11.08
**A0A0G9FBB8**	Ribosome hibernation promoting factor	36.29	13.83	21.7	5.54
**A0A0G9FBT8**	DUF5590 domain-containing protein	35.24	5.88	17.0	10.13
**A0A0G9FBL7**	Large ribosomal subunit protein bL19	34.74	8.47	13.6	11.43
**A0A0L7Y0D5**	DUF308 domain-containing protein	34.63	9.09	22.9	10.11
**A0A0G9FBT3**	DUF1129 domain-containing protein	34.59	4.56	26.6	9.88
**A0A165NTV2**	Plasmid replication initiation protein	34.09	1.96	36.1	9.57
**A0A0G9FH87**	DUF805 domain-containing protein	33.21	11.57	14.2	8.56
**A0A0R2G235**	Small ribosomal subunit protein uS2	33.11	7.63	29.7	5.41
**A0A0G9F6I2**	2.3-bisphosphoglycerate-dependent phosphoglycerate mutase	32.48	20.87	26.1	5.07
**A0A0G9GY95**	Large ribosomal subunit protein uL1	32.21	4.37	24.8	8.19
**A0A165RF64**	Phospho-sugar mutase	31.98	3.13	63.5	5.11
**A0A0G9FFS3**	Small ribosomal subunit protein uS17	31.78	12.36	10.3	10.10
**A0A151G3S1**	Glycerol phosphate lipoteichoic acid synthase	30.70	1.29	78.9	9.29
**A0A0G9GUK5**	Small ribosomal subunit protein uS13	29.46	16.53	13.6	10.39
**A0A0G9FAC6**	Large ribosomal subunit protein uL14	28.14	27.87	13.1	9.91
**A0A165EZP7**	Uncharacterized protein	27.75	10.71	12.9	7.43
**A0A0G9FIW0**	Glucosamine-6-phosphate deaminase	27.67	6.75	25.9	5.08
**A0A151G865**	Membrane protein 6-pyruvoyl-tetrahydropterin synthase-related domain-containing protein	26.93	1.02	66.8	10.18
**A0A0G9FCX0**	Thioredoxin	26.82	14.56	11.3	4.54

**A0A836NNX2**	Uncharacterized protein	26.44	6.07	23.7	9.33
**A0A0G9F9U2**	Phosphate-binding protein	26.35	4.12	31.5	9.82
**A0A0G9FBQ2**	Exonuclease SbcC	25.99	5.76	34.5	5.12
**A0A0R2G5D5**	Single-stranded DNA-binding protein	25.96	5.23	17.2	6.38
**A0A151G8Z7**	LCP family protein	24.99	3.02	43.6	9.82
**A0A0G9FBY5**	Small ribosomal subunit protein uS9	24.80	16.15	14.6	10.39
**A0A0L7Y0C1**	C-terminal processing peptidase	24.08	2.44	53.2	9.82
**A0A0L7Y2W3**	CNT family concentrative nucleoside transporter	23.19	6.56	46.3	5.52
**A0A0R2GAR4**	ATP-dependent zinc metalloprotease FtsH	22.51	0.82	78.6	5.63
**A0A0G9FC86**	ABC-type glycine betaine transport system. substrate-binding domain	22.19	2.25	34.8	9.45
**A0A0G9FC74**	Dihydrolipoyl dehydrogenase	21.82	5.18	48.2	5.39
**A0A0R2GD57**	Nucleoside 2-deoxyribosyltransferase	21.47	7.96	12.6	5.22
**A0A0G9FET7**	30S ribosomal protein S1	21.25	4.66	47.1	4.88
**A0A0G9F6F8**	Protein translocase subunit SecE	20.31	18.03	7.5	10.26
**A0A151G4G0**	Endolytic murein transglycosylase	20.13	2.24	39.6	9.57

Table 2. Mass spectrometry identification of proteins from *Lacticaseibacillus rhamnosus* PCM 489 extracellular vesicles (EVs).

Accession Number	Protein Description	Score	Sequence Coverage [%]	Molecular Mass [kDa]	pI
A0A7X2J656	C40 family peptidase	5181.08	61.90	40.9	8.60
A0A7Z9DRM1	Lysozyme	3501.69	65.27	74.5	9.04
A0A853J739	S8 family serine peptidase	3395.36	44.64	204.5	5.47
A0A180C7B5	CHAP domain-containing protein	3057.94	41.35	42.6	7.52
A0A853J752	Fibrinogen-binding protein (Fragment)	2393.00	45.45	103.1	4.73
A0A249DJ24	Cell surface protein	2195.61	23.18	154.6	4.81
A0A508YHN0	S8 family serine peptidase	2038.58	32.46	155.6	5.35
A0A7X2J721	LysM peptidoglycan-binding domain-containing protein	1922.37	45.75	128.2	6.83
A0A508Z0M7	Hydrolase	1379.41	42.77	34.2	7.47
A0A0E3CQZ4	ABC transporter substrate-binding protein	1124.95	51.49	48.2	9.52
A0A0D6UAI2	LCP family protein	1034.19	56.59	40.2	9.54
A0A853J2C3	KxYKxGKxW signal peptide domain-containing protein	942.60	12.80	322.6	5.41
A0A171J8Q8	N-acetylmuramoyl-L-alanine amidase	773.58	51.59	47.3	8.85
A0A0E3CRJ7	Glyceraldehyde-3-phosphate dehydrogenase	771.15	53.53	36.7	5.83
A0A0J6UTL1	Oligopeptide ABC transporter substrate-binding protein	767.88	41.88	66.4	9.45
A0A508Z3B1	serine-type D-Ala-D-Ala carboxypeptidase	742.74	41.51	46.9	9.51
A0A2A5L5E7	Pyridoxamine 5'-phosphate oxidase family protein	672.99	67.67	14.7	5.29
A0A249DBM7	Glycerol phosphate lipoteichoic acid synthase	610.13	31.13	77.9	8.57
A0A0E3CLJ0	FMN-binding domain-containing protein	609.44	50.99	32.7	9.47
A0A0E3CNA6	Tagatose 1.6-diphosphate aldolase]	587.81	59.04	36.3	5.27
A0A508YXM9	Peptide ABC transporter substrate-binding protein	553.21	35.79	61.1	9.67
A0A508YP55	Peptidoglycan binding domain-containing protein	544.18	48.68	50.1	8.51
A0A508Z384	PBP1A family penicillin-binding protein	536.07	22.11	83.4	9.10
A0A9Q5ETC5	Carboxypeptidase	508.25	21.23	99.2	9.09
A0A249DAG4	Glycoside hydrolase	500.58	21.72	48.9	6.93
A0A7X2M372	ABC transporter substrate-binding protein	492.71	48.76	34.0	9.70
A0A7X2J725	LysM peptidoglycan-binding domain-containing protein	492.42	35.37	51.4	7.72
A0A7Y7QJN1	Sortase	482.51	35.33	30.6	9.54
A0A0E3CNL5	ABC transporter permease subunit	481.84	28.57	58.3	9.51
A0A7S7FQ36	Sortase	471.43	35.33	30.6	9.54
A0A180BLS7	Pyruvate oxidase	457.18	23.46	64.0	5.20
A0A0E3D3H8	Class A sortase	455.81	36.05	25.8	9.82
A0A171J5F2	Serine protease	448.62	40.32	45.2	6.18
A0A180AC74	ABC transporter substrate-binding protein	409.63	41.18	40.6	6.67
A0A249DEM0	Acyltransferase	403.59	30.71	26.5	9.36
A0A249DEN2	Peptide ABC transporter substrate-binding protein	399.83	28.94	59.4	9.51
A0A0D6UDC7	Foldase protein PrsA	399.33	29.00	33.5	9.64
A0A7Y7UJC2	Zinc-ribbon domain-containing protein	386.83	19.04	42.9	5.39
A0A7Y7QDV6	Peptide ABC transporter substrate-binding protein	375.33	23.81	60.3	9.47
A0A0E3CRX8	Flagellar hook-length control protein FliK	361.75	31.13	32.5	5.11
A0A0D6U5I1	LCP family protein	346.33	17.61	39.1	9.52
A0A853J0Q3	Efflux RND transporter periplasmic adaptor subunit	327.90	31.55	38.8	9.09
A0A249DEM6	Alkaline phosphatase	323.27	16.09	84.0	7.20
A0A5P5ZEZ3	L-lactate dehydrogenase	319.29	26.69	35.5	5.45
A0A0E3CMF0	Uncharacterized protein 4	309.53	52.87	18.9	9.77
A0A249DG42	Peptidase	297.99	26.61	34.7	9.58
A0A7S7FS31	Extracellular solute-binding protein	279.95	24.06	46.4	9.63
A0A0J6WMB6	Calcium ABC transporter ATPase	279.49	24.80	41.7	7.46
A0A171JAF8	Enolase	279.30	19.59	47.1	4.79
A0A809N520	Chaperonin GroEL	274.96	22.45	55.8	5.03
A0A508YI06	FliK family flagellar hook-length control protein	272.52	29.60	42.1	5.55
A0A7X2J3K8	Lipoprotein	262.44	21.05	30.1	4.96

**A0A0D6UBJ9**	Elongation factor Tu	260.49	26.77	43.5	4.93
**A0A249DE53**	Phosphoenolpyruvate-protein phosphotransferase	258.40	13.59	63.2	5.19
**A0A249DGI4**	Alpha/beta hydrolase	257.42	20.75	28.7	8.41
**A0A508YWS0**	DUF5067 domain-containing protein	255.46	56.84	20.8	8.79
**A0A508Z1W1**	MucBP domain-containing protein	242.66	17.35	46.5	9.22
**A0A180BN67**	Cell division protein FtsI	237.17	16.53	77.4	9.14
**A0A171J6U3**	Large ribosomal subunit protein bL12	221.74	50.00	12.6	4.63
**A0A508YZA0**	Beta-lactamase family protein	213.31	22.02	42.8	9.52
**A0A508YL94**	Cell surface protein	210.43	27.59	33.5	9.61
**A0A508Z6Z5**	Extracellular protein	208.21	19.62	33.9	9.44
**A0A7X2J4N6**	ATP synthase subunit b	199.76	38.27	17.7	7.40
**A0A508YJC4**	Discoidin domain-containing protein	195.74	4.91	195.0	6.61
**A0A853J132**	Uncharacterized protein	191.40	32.00	13.7	10.33
**A0A180BKL5**	PTS sugar transporter	188.94	52.88	11.3	5.57
**A0A508YQD0**	Cysteine synthase	185.89	20.71	32.6	5.54
**A0A249DB01**	Aminopeptidase	182.79	8.06	94.5	5.20
**A0A7X2J5C7**	KxYKxGKxW signal peptide domain-containing protein	176.69	5.15	93.0	6.48
**A0A508Z806**	YxeA family protein	176.00	39.84	14.2	9.63
**A0A249DAC9**	Hydrolase	173.70	24.78	23.3	5.08
**A0A0D6U7E1**	Acyltransferase	171.10	20.67	33.1	9.29
**A0A249DIL7**	Matrix-binding protein	161.20	2.76	245.6	5.38
**A0A0D6U5T3**	Helix-turn-helix domain-containing protein	156.67	25.58	14.0	6.10
**A0A249DHV7**	Polysaccharide lyase	153.86	3.53	112.5	7.02
**A0A2A5L7N8**	2.3-bisphosphoglycerate-dependent phosphoglycerate mutase	152.60	27.95	25.9	5.52
**A0A6N3C390**	Glutamine-binding periplasmic protein	151.83	8.32	50.8	9.61
**A0A7S7FQT0**	Small ribosomal subunit protein uS11	149.59	21.71	13.8	11.63
**A0A0D6U8T5**	Small ribosomal subunit protein uS12	148.43	23.19	15.2	11.40
**A0A0J6UPB2**	Triosephosphate isomerase	144.57	29.88	26.8	5.01
**A0A9Q5ELE3**	Peptidoglycan-binding protein LysM	144.37	16.78	50.3	6.58
**A0A873ZER7**	Cell surface protein	143.01	24.14	33.5	9.61
**A0A179YR60**	Pyruvate kinase	142.22	9.52	62.8	5.43
**A0A7S7FMV4**	Alpha-glycerophosphate oxidase	136.69	11.69	67.0	5.20
**A0A0D6U9Y8**	Large ribosomal subunit protein bL17	135.37	31.75	14.2	10.10
**A0A0E3CQF7**	Peptide ABC transporter substrate-binding protein	132.28	13.15	59.8	9.38
**A0A0J6USA8**	Lipoprotein	129.70	14.49	30.6	9.88
**A0A249DFM4**	WxL domain-containing protein	125.76	20.00	20.6	4.72
**A0A508YKT1**	FIVAR domain-containing protein	123.97	7.67	81.6	6.62
**A0A508YLS1**	FIVAR domain-containing protein	121.60	4.42	160.5	5.95
**A0A508Z2F0**	Extracellular protein	117.65	13.30	21.0	9.11
**A0A249DEQ7**	Endolytic murein transglycosylase	117.36	8.88	42.1	9.61
**A0A0E3CQT4**	6-phosphogluconolactonase	116.95	17.63	37.6	5.20
**A0A171J9Z4**	Glucose-6-phosphate isomerase	116.48	24.44	49.3	5.12
**A0A249DGC2**	Lipoprotein	112.74	9.51	30.0	9.28
**A0A2A5L381**	Large ribosomal subunit protein uL2	111.64	12.23	30.4	10.30
**A0A0E3CQW6**	Large ribosomal subunit protein bL20	109.96	21.19	13.5	11.47
**A0A2A5L2Z6**	Large ribosomal subunit protein uL4	109.04	19.81	22.4	9.92
**A0A1Y0E116**	Lipase	108.69	9.25	30.7	9.58
**A0A2A5L7A9**	Small ribosomal subunit protein uS2	106.51	15.65	29.6	5.38
**A0A2A5L336**	Small ribosomal subunit protein uS5	106.40	30.54	17.5	7.52
**A0A5R9D563**	ABC transporter substrate-binding protein	104.85	5.59	46.8	9.60
**A0A809NCA4**	Large ribosomal subunit protein uL22	104.19	25.64	12.7	9.99
**A0A0D6U8P4**	Large ribosomal subunit protein uL16	104.17	19.44	16.0	10.74
**A0A249DIB0**	ABC transporter permease subunit	102.06	5.76	52.9	9.67
**A0A171J972**	Lipoprotein	100.84	6.64	30.1	10.24
**A0A0E3CQ75**	Small ribosomal subunit protein uS7	99.63	25.00	17.9	9.89
**A0A0E3CPH7**	Large ribosomal subunit protein uL5	96.83	13.33	20.2	8.29

**A0A249DCV7**	Penicillin-binding protein	93.42	6.44	76.8	9.63
**A0A249DC63**	Branched-chain amino acid ABC transporter substrate-binding protein	86.76	13.35	41.5	9.58
**A0A0E3D3X6**	Carboxypeptidase	83.46	9.12	74.7	9.17
**A0A249DEC8**	ABC transporter permease	82.64	4.12	52.7	9.54
**A0A0D6U4B9**	Large ribosomal subunit protein uL11	80.90	12.06	14.9	8.91
**A0A249DE67**	Phosphoglucomutase	79.99	7.30	63.6	5.44
**A0A171JA73**	Large-conductance mechanosensitive channel	78.38	14.75	13.6	9.70
**A0A0D6U7B4**	Aminopeptidase	76.66	8.93	50.7	5.36
**A0A0E3CN98**	NAD(P)/FAD-dependent oxidoreductase	76.45	3.48	70.8	9.06
**A0A171J8M4**	Chaperone protein DnaK	76.24	3.23	67.2	4.98
**A0A7S7FRV4**	Large ribosomal subunit protein uL29	75.26	20.31	7.6	9.99
**A0A0E3D054**	YkyA family protein	73.90	12.62	23.5	5.64
**Q58Z11**	Capsular polysaccharide biosynthesis protein CpsC	73.72	8.55	34.0	9.66
**A0A171J992**	Trigger factor	73.43	4.48	49.8	4.70
**A0A0E3CNN1**	30S ribosomal protein S1	72.96	5.96	47.1	5.27
**A0A2A5L9I8**	Small ribosomal subunit protein bS20	71.85	17.86	9.4	10.70
**A0A0J7A425**	Glycerol kinase	71.16	3.76	55.6	5.12
**A0A0D6U6M1**	Protein DltD	70.50	5.20	48.0	9.57
**A0A2A5L320**	Large ribosomal subunit protein bL9	70.18	21.19	16.9	9.83
**A0A0D6U4U7**	DUF4430 domain-containing protein	68.15	20.77	14.2	10.40
**A0A508YUC3**	PTS glucose transporter subunit IIA	67.79	1.80	70.3	7.55
**A0A0E3CNY7**	Phosphate-binding protein	66.52	8.97	31.9	9.42
**A0A2A5L393**	Large ribosomal subunit protein uL6	65.13	8.52	19.3	9.67
**A0A7S7FQT8**	Large ribosomal subunit protein uL14	64.84	18.85	13.0	10.05
**A0A1Y0DVX4**	PTS fructose transporter subunit IIC	63.06	5.26	25.8	6.68
**A0A180C927**	Signal peptidase I	63.03	14.07	22.8	9.96
**A0A809N128**	Large ribosomal subunit protein uL23	62.20	20.00	11.5	9.92
**A0A7S7JHJ9**	Large ribosomal subunit protein uL18	61.94	10.92	13.0	10.05
**A0A171J7I5**	Glutamate ABC transporter substrate-binding protein	61.40	5.11	29.7	9.76
**A0A0E3D3P6**	Phosphoglycerate kinase	60.10	7.58	42.2	5.95
**A0A7Y7QE53**	YhgE/Pip domain-containing protein	59.58	5.16	99.0	9.03
**A0A249DAX2**	Cell division protein FtsI	59.20	1.36	70.8	9.45
**A0A2A5L2L1**	Bifunctional 2-keto-4-hydroxyglutarate aldolase/2-keto-3-deoxy-6-phosphogluconate aldolase	59.17	8.33	22.4	5.24
**A0A6N2ZNF9**	3-oxoacyl-[acyl-carrier-protein] synthase 2	58.32	9.11	42.3	5.35
**A0A0D6U4U2**	Large ribosomal subunit protein uL1	58.25	14.41	24.5	9.28
**A0A0M2G0T3**	FAD-dependent oxidoreductase	58.20	4.61	49.3	5.22
**A0A0D6UE79**	Large ribosomal subunit protein bL21	57.92	13.59	11.3	9.45
**A0A171J3P6**	Dihydroxyacetone kinase	57.03	11.92	20.6	6.81
**A0A0J6UYA1**	Glycine/betaine ABC transporter substrate-binding protein	56.36	9.06	34.7	9.23
**A0A0E3CNP0**	Uncharacterized protein	55.77	13.33	23.7	8.82
**A0A0D6U8S2**	Large ribosomal subunit protein uL13	55.64	14.86	16.6	9.63
**A0A2A5L6V2**	Cell division ATP-binding protein FtsE	54.09	9.65	25.5	5.53
**A0A249DD67**	endopeptidase La	53.04	3.59	39.2	9.91
**A0A180BFG9**	FAD:protein FMN transferase	52.33	4.26	38.5	9.42
**A0A0D6UB62**	Cysteine desulfurase	52.09	11.50	12.7	9.41
**A0A179WBK1**	Extracellular protein	51.33	7.69	25.5	10.14
**A0A809NA35**	Small ribosomal subunit protein uS13	51.00	5.79	13.5	10.49
**A0A0E3D3B9**	Large ribosomal subunit protein uL3	50.60	8.10	22.6	9.82
**A0A1Y0DZ69**	Uncharacterized protein	50.07	4.08	35.3	7.55
**A0A249DB19**	WxL domain-containing protein	49.39	4.05	22.4	4.94
**A0A809N776**	Phage nuclease	47.47	5.69	27.3	6.81
**A0A249DGN9**	D-alanyl-D-alanine carboxypeptidase	47.29	12.50	26.3	9.31
**A0A180BGV5**	Metal ABC transporter substrate-binding protein	46.86	7.21	33.3	9.09
**A0A0D6UAV8**	Small ribosomal subunit protein uS4	45.56	9.36	23.3	9.76
**A0A0E3D3E6**	inorganic diphosphatase	45.52	9.68	33.5	4.93
**A0A0D6U5M7**	3-hydroxyacyl-[acyl-carrier-protein] dehydratase FabZ	45.31	15.75	15.9	8.13

**A0A249DGB3**	Capsular polysaccharide biosynthesis protein CpsC	44.76	4.35	24.7	9.47
**Q9FDJ7**	Putative glycosyltransferase (Fragment)	44.06	5.47	23.5	8.62
**A0A508YUJ8**	ABC transporter ATP-binding protein	44.01	3.57	27.2	5.95
**A0A249DIG7**	Nicotinate phosphoribosyltransferase	43.04	2.52	52.3	6.37
**A0A249DCA1**	Serine O-acetyltransferase	43.00	3.27	32.1	7.36
**A0A0D6U787**	LemA family protein	42.55	4.46	22.1	9.51
**A0A249DEM2**	Peptide ABC transporter ATP-binding protein	42.39	2.36	32.8	6.98
**A0A171J468**	PTS fructose transporter subunit IID	42.35	8.42	29.8	9.88
**A0A171J6N2**	dUTP diphosphatase	42.24	6.15	20.5	9.23
**A0A7Y7UHH7**	Zinc ribbon domain-containing protein	42.07	5.01	38.3	9.54
**A0A249DEK9**	Tyrosine-protein kinase CpsD	42.02	4.25	22.5	5.55
**A0A0D6U5D8**	Co-chaperonin GroES	41.98	9.68	10.0	5.01
**A0A2A5L2P2**	PTS mannose transporter subunit IIAB	41.83	3.75	17.3	8.40
**A0A0E3CPJ8**	Small ribosomal subunit protein uS9	41.50	7.69	14.3	10.59
**A0A508YVM5**	Matrixin family metalloprotease	41.44	4.84	26.5	8.90
**A0A180A1S0**	Permease IIC component	40.97	2.42	49.3	9.00
**A0A249DES4**	6-phosphogluconate dehydrogenase. decarboxylating	40.69	1.91	52.0	5.50
**A0A2A5L9L1**	DNA-binding protein HU	39.87	30.77	9.5	9.85
**A0A0D6UFA6**	Class II fructose-1.6-bisphosphate aldolase	39.56	3.41	31.5	5.14
**H6SHW1**	RNA polymerase (Fragment)	38.89	10.00	10.4	8.59
**A0A7Y7QDD7**	Zinc-ribbon domain-containing protein	38.34	6.03	36.8	7.39
**A0A0D6UC46**	Small ribosomal subunit protein bS21	38.26	13.79	7.0	10.71
**A0A0J6TQ18**	ATP synthase subunit alpha	37.72	4.13	55.2	5.38
**A0A249DH01**	Manganese ABC transporter substrate-binding protein	37.45	3.19	35.0	9.28
**A0A0J6UFS9**	5-bromo-4-chloroindolyl phosphate hydrolysis family protein	37.11	4.55	24.8	6.55
**A0A180BGT4**	Two-component system regulatory protein YycI	36.96	4.91	29.9	9.39
**A0A508Z8T5**	DNA-entry nuclease	35.31	2.83	25.7	10.24
**A0A0D6U666**	DUF1049 domain-containing protein	34.85	12.04	11.8	9.99
**A0A179XLR4**	Foldase protein PrsA	34.84	3.33	33.1	9.80
**A0A180B9V3**	Hsp20 family protein	34.32	8.22	16.6	5.03
**A0A249DD20**	Molecular chaperone	34.05	8.23	17.8	5.10
**A0A809N821**	Uncharacterized protein	33.94	5.19	15.1	9.50
**A0A0J6XR26**	AsparaGIne synthase (Glutamine-hydrolyzing)	33.55	1.26	73.3	6.44
**A0A7X2M1V9**	Leucine-rich repeat protein	33.43	1.43	109.1	5.00
**A0A249DCT8**	D-ribose ABC transporter substrate-binding protein	33.29	4.39	33.4	9.20
**A0A249DHG0**	Small ribosomal subunit protein uS17	33.24	21.74	8.0	9.79
**A0A809NA46**	Small ribosomal subunit protein uS8	32.83	17.42	14.8	9.58
**A0A0E3CPD9**	Small ribosomal subunit protein uS3	32.79	8.18	24.9	9.98
**A0A2A5L6V8**	Probable transcriptional regulatory protein BGK71_04670	32.69	5.35	27.1	4.84
**A0A7Y7QI03**	Transposase	31.18	1.78	39.3	9.91
**A0A2A5L886**	Glycosyl transferase family 2	31.01	2.07	27.2	6.09
**A0A180CM60**	Streptococcin A-M57	30.70	5.38	20.7	7.93
**A0A179X3E8**	Alpha/beta hydrolase	30.40	4.52	34.6	9.72
**A0A249DG83**	ATP-dependent zinc metalloprotease FtsH	30.07	1.68	78.2	5.48
**A0A0E3CL48**	Uncharacterized protein	30.05	17.84	20.1	4.98
**A0A249DGA6**	Galactose mutarotase	29.77	3.41	32.6	5.27
**A0A171JAH8**	Thioredoxin reductase	29.61	3.81	34.2	5.55
**A0A0J6X4K6**	Phosphate-binding protein	29.30	6.25	30.8	9.77
**A0A249DHD7**	Metal ABC transporter substrate-binding protein	29.27	5.00	33.4	9.25
**A0A0E3CL79**	ATP synthase subunit delta	28.76	4.97	19.4	8.40
**A0A0D6U9U8**	ATP-dependent Clp protease ATP-binding subunit	28.24	1.56	91.6	6.58
**A0A5P5ZAS3**	CDP-diacylglycerol--glycerol-3-phosphate 3-phosphatidyltransferase	28.21	5.10	21.9	9.74
**A0A249DA30**	Cell surface protein	27.45	4.48	28.4	5.76
**A0A2A5L730**	Rhodanese-like domain-containing protein	27.20	6.67	15.9	9.92
**A0A0E3CQC0**	ATP-dependent 6-phosphofructokinase	27.15	2.82	34.2	6.05
**A0A0E3CQ27**	L-lactate dehydrogenase	26.76	2.66	32.5	5.53

**A0A0J6U736**	Membrane protein insertase YidC	26.52	4.55	36.5	9.61
**A0A7S7JGT6**	S-ribosylhomocysteine lyase	25.75	5.73	17.2	6.28
**A0A0J6UYU8**	Membrane protein	25.54	4.22	18.2	9.99
**A0A7S7FPQ2**	Dihydrolipoamide acetyltransferase component of pyruvate dehydrogenase complex	25.19	3.17	46.8	5.20
**A0A0E3CLE4**	DJ-1/PfpI/YhbO family deglycase/protease	23.86	22.62	18.4	5.08
**A0A0E3CQD1**	Large ribosomal subunit protein uL10	23.38	7.14	18.2	5.15
**A0A0E3CPP5**	Amidase	23.27	4.35	22.8	5.38
**A0A7Y7QI94**	LacI family DNA-binding transcriptional regulator	23.20	1.45	38.5	6.44
**A0A0E3CMX6**	Dihydrolipoyl dehydrogenase	23.06	1.93	49.1	5.91
**A0A7S7FRT8**	Small ribosomal subunit protein uS19	22.59	8.60	10.6	10.13
**A0A0D6UCL0**	Small ribosomal subunit protein bS16	22.10	7.69	10.5	10.24
**A0A2A5L7B6**	Elongation factor Ts	21.68	7.17	31.6	5.11
**A0A7S7FRS9**	UTP--glucose-1-phosphate uridylyltransferase	21.49	3.62	33.8	6.27
**A0A0J6ULP2**	Phosphate ABC transporter ATP-binding protein	21.06	4.04	30.8	6.18
**A0A6N3BY57**	YxeA family protein	20.67	12.70	14.5	9.17
**A0A249DFB4**	Surfactant protein C. propeptide	20.52	3.59	26.7	4.70
**A0A249DGN0**	Cellulose synthase	20.48	1.17	75.2	9.63
**Accession Number**	**Protein Description**	**Score**	**Sequence Coverage [%]**	**Molecular Mass [kDa]**	**pI**
**GI|258540465**	fibrinogen-binding protein	428.00	17.00	105.9	4.56
**GI|385836102**	LPXTG-motif cell wall anchor domain-containing protein	428.00	15.00	116.5	4.67
**GI|258509277**	hypothetical protein LGG_02282	428.00	12.00	135.8	4.60
**GI|385828913**	putative cell surface protein	428.00	11.00	155.1	4.61
**GI|491951815**	fibrinogen-binding protein. partial	428.00	23.00	79.6	4.52
**GI|491959236**	fibrinogen-binding protein. partial	428.00	19.00	94.6	4.66
**GI|258540184**	lyzozyme M1	261.00	21.00	74.4	9.03
**GI|492000601**	Lyzozyme M1 (1.4-beta-N-acetylmuramidase)	261.00	21.00	74.4	9.03
**GI|491996440**	hypothetical protein	242.00	27.00	40.8	8.81
**GI|258507221**	pyridoxine 5'-phosphate oxidase V-like favin-nucleotide-binding protein	152.00	65.00	14.7	5.08
**GI|258507928**	glyceraldehyde-3-phosphate dehydrogenase	122.00	32.00	36.7	5.53
**GI|258540404**	hypothetical protein LC705_02213	102.00	15.00	34.2	6.84
**GI|491959389**	hydrolase	102.00	14.00	37.3	9.22
**GI|479146350**	23S rRNA m(5)U-1939 methyltransferase	100.00	7.00	53.1	6.00
**GI|58337987**	hypothetical protein LBA1729	99.00	4.00	25.9	9.02
**GI|410666775**	60 kDa chaperonin	99.00	5.00	58.4	5.10
**GI|258507026**	surface antigen	98.00	17.00	42.6	6.97
**GI|258538216**	surface antigen	98.00	17.00	42.6	6.54
**GI|491951590**	hypothetical protein	98.00	17.00	42.6	6.54
**GI|183393070**	surface antigen	88.00	29.00	17.3	9.10
**GI|258507825**	sulfatase	81.00	6.00	77.9	8.53
**GI|258539015**	sulfatase	81.00	6.00	77.9	8.50
**GI|491960874**	glycerol phosphate lipoteichoic acid synthase	81.00	6.00	78.7	8.66
**GI|491997881**	glycerol phosphate lipoteichoic acid synthase	81.00	6.00	78.7	8.53
**GI|492013601**	Lipoteichoic acid synthase LtaS Type IIa	81.00	6.00	77.9	8.36
**GI|495221972**	elongation factor Tu domain-containing protein. partial	74.00	14.00	21.0	5.37
**GI|331268633**	23S rRNA (uracil-5-)-methyltransferase RumA	71.00	1.00	56.4	7.01
**GI|258508080**	glucose-6-phosphate isomerase	68.00	7.00	49.3	5.00
**GI|258539353**	glucose-6-phosphate isomerase	68.00	7.00	49.3	5.00
**GI|491953476**	glucose-6-phosphate isomerase	68.00	7.00	54.0	5.29
**GI|491992997**	glycoside hydrolase	68.00	14.00	49.6	6.41
**GI|489645853**	30S ribosomal protein S21. partial	68.00	49.00	7.9	11.16
**GI|491996353**	peptidoglycan-binding protein LysM	67.00	3.00	130.9	6.46
**GI|491999471**	peptidoglycan-binding protein LysM	67.00	3.00	128.1	6.28
**GI|258507319**	cell wall-associated glycoside hydrolase (NLP/P60 protein)	66.00	17.00	49.7	6.41

**GI|258538501**	cell wall-associated glycoside hydrolase (NLP/P60 protein)	66.00	17.00	49.3	6.41
**GI|491999634**	2-hydroxy-6-oxo-6-phenylhexa-2.4-dienoate hydrolase	66.00	17.00	48.4	7.71
**GI|183393106**	trypsin-like serine protease	64.00	8.00	38.4	5.22
**GI|258509801**	serine protease	64.00	7.00	45.2	5.81
**GI|491945612**	serine protease	64.00	7.00	45.2	6.25
**GI|491998718**	serine protease	64.00	7.00	45.2	5.81
**GI|89893756**	hypothetical protein DSY1010	64.00	7.00	15.4	4.79
**GI|56419350**	oligopeptide ABC transporter ATP-binding protein	62.00	3.00	35.5	7.74
**GI|446651568**	hypothetical protein	59.00	2.00	53.9	7.59
**GI|489680568**	hypothetical protein	59.00	31.00	6.2	10.75
**GI|493766535**	molecular chaperone GroEL	59.00	6.00	57.7	4.86
**GI|170759773**	hypothetical protein CLK_0109	58.00	6.00	13.2	10.01
**GI|302669874**	glycosyl transferase 2	58.00	2.00	37.9	5.04
**GI|491694802**	acyl-CoA hydrolase	58.00	4.00	20.5	8.45
**GI|489710067**	cell wall surface anchor protein	57.00	3.00	53.7	5.29
**GI|490830907**	polyribonucleotide nucleotidyltransferase	57.00	4.00	75.0	5.09
**GI|489351640**	LtrC-like protein	57.00	15.00	8.4	9.65
**GI|496710386**	phosphate transport regulator	57.00	4.00	24.2	4.65
**GI|493634418**	proteinase	56.00	3.00	26.4	6.36
**GI|495794709**	L-aspartate oxidase	55.00	6.00	56.7	7.29
**GI|374298063**	30S ribosomal protein S14	54.00	21.00	7.2	10.54
**GI|497881057**	O-succinylbenzoate synthase	54.00	8.00	42.0	5.40
**GI|339627472**	chaperone protein DnaK	54.00	6.00	68.3	5.05
**GI|496351263**	elongation factor Tu	53.00	3.00	44.3	5.21
**GI|374997042**	exopolyphosphatase-like protein	52.00	4.00	35.8	5.78
**GI|81429365**	50S ribosomal protein L5	51.00	8.00	20.2	9.34
**GI|104773557**	50S ribosomal protein L6	48.00	3.00	19.3	9.66
**GI|258507047**	2-dehydro-3-deoxyphosphogluconate aldolase	47.00	5.00	22.4	5.03
**GI|407472821**	collagen adhesion protein	46.00	1.00	192.3	4.74
**GI|404282345**	ABC transporter ATP-binding protein	46.00	2.00	34.6	5.59
**GI|78043711**	LysM domain-containing protein	45.00	1.00	55.6	5.81
**GI|495774022**	oxidoreductase. FAD-binding protein	45.00	2.00	48.9	4.74
**GI|56962937**	cell surface protein	43.00	1.00	310.7	4.00
**GI|258508046**	TetR family transcriptional regulator	43.00	3.00	23.0	6.32
**GI|319891781**	enolase	42.00	5.00	47.0	4.50
**GI|491523508**	transposase. partial	42.00	3.00	27.3	8.75
**GI|184154568**	hypothetical protein LAF_0092	42.00	6.00	38.7	9.57
**GI|77964181**	ATP synthase subunit beta	41.00	4.00	50.4	5.01
**GI|493781614**	elongation factor Tu	40.00	8.00	43.5	4.99
**GI|254557145**	ATP synthase F0F1 subunit alpha	37.00	4.00	54.6	4.95
**GI|489796080**	transposase ISL3	36.00	9.00	50.3	9.71
**GI|311067500**	penicillin-binding protein 1	35.00	5.00	79.0	8.67
**GI|511478511**	surface antigen	30.00	10.00	41.6	8.93
**GI|498310663**	molecular chaperone GroES	29.00	17.00	10.2	4.98
**GI|58337164**	aspartate-semialdehyde dehydrogenase	27.00	2.00	38.7	5.19
**GI|116496026**	tagatose 1.6-diphosphate aldolase	26.00	6.00	36.3	5.25
**GI|258507660**	tagatose 1.6-diphosphate aldolase	26.00	6.00	36.4	5.24
